# Acoustofluidic rotational tweezing enables high-speed contactless morphological phenotyping of zebrafish larvae

**DOI:** 10.1038/s41467-021-21373-3

**Published:** 2021-02-18

**Authors:** Chuyi Chen, Yuyang Gu, Julien Philippe, Peiran Zhang, Hunter Bachman, Jinxin Zhang, John Mai, Joseph Rufo, John F. Rawls, Erica E. Davis, Nicholas Katsanis, Tony Jun Huang

**Affiliations:** 1 Department of Mechanical Engineering and Material Science, Duke University, Durham, NC USA; 2 Center for Human Disease Modeling, Duke University Medical Center, Durham, NC USA; 3 Alfred E. Mann Institute for Biomedical Engineering, University of Southern California, Los Angeles, CA USA; 4 Department of Molecular Genetics and Microbiology, Duke University, Durham, NC USA; 5 Advanced Center for Translational and Genetic Medicine (ACT-GeM), Stanley Manne Children’s Research Institute, Ann & Robert H. Lurie Children’s Hospital of Chicago, Chicago, IL USA; 6 Department of Pediatrics, Feinberg School of Medicine, Northwestern University, Chicago, IL USA

**Keywords:** 3-D reconstruction, Biotechnology, Engineering, Acoustics

## Abstract

Modern biomedical research and preclinical pharmaceutical development rely heavily on the phenotyping of small vertebrate models for various diseases prior to human testing. In this article, we demonstrate an acoustofluidic rotational tweezing platform that enables contactless, high-speed, 3D multispectral imaging and digital reconstruction of zebrafish larvae for quantitative phenotypic analysis. The acoustic-induced polarized vortex streaming achieves contactless and rapid (~1 s/rotation) rotation of zebrafish larvae. This enables multispectral imaging of the zebrafish body and internal organs from different viewing perspectives. Moreover, we develop a 3D reconstruction pipeline that yields accurate 3D models based on the multi-view images for quantitative evaluation of basic morphological characteristics and advanced combinations of metrics. With its contactless nature and advantages in speed and automation, our acoustofluidic rotational tweezing system has the potential to be a valuable asset in numerous fields, especially for developmental biology, small molecule screening in biochemistry, and pre-clinical drug development in pharmacology.

## Abstract (web summary)

Existing methods of zebrafish phenotyping rely on contact-based processes. Here the authors report on an acoustofluidic-based platform which performs contactless specimen rotation, that results in multispectral images for rapid morphological phenotyping of zebrafish larvae.

## Introduction

The zebrafish (*Danio rerio*) is a widely used vertebrate model organism for biological, medical, and pathology research^1,2^. These vertebrates have been especially useful for in vivo chemical screening for small molecule drug discovery^2,3^, in which the visualization and analysis of the resulting phenotypes are widely used to explore the effects of drug candidates on developing and mature animals. Owing to their high reproduction rate, large-scale phenotyping-based studies can be performed to obtain statistically significant measurements and reliable conclusions^4^, which requires an efficient manipulation and analysis platform. Considering the significant potential that zebrafish hold for rapid drug screening and disease evaluation, it is important to develop functional platforms that provide clear visualization and accurate analysis for high-throughput phenotypic evaluations of zebrafish larvae^5^.

Although numerous innovations in optical imaging techniques have been made, solely using an imaging technique is often insufficient for efficiently phenotyping zebrafish larvae^6–8^. This is not only due to the limited throughput, but also the obstruction of the internal regions of interest by organs (e.g., eyes, yolk sac) or skin pigmentation^9^. As a result, researchers have devoted significant effort to developing manipulation techniques that enable precise orientation of the zebrafish, such that a desired angular alignment can be achieved to allow for the visualization of obscured features^10–15^. For example, the zebrafish immobilization and orientation method of agarose/gel-based confinement^10,16^ fixes zebrafish for optimal observation at the desired viewing angles. However, this contact-based method relies on a tedious fixing process, wherein the gelation process may induce external effects on the zebrafish’s natural physiological characteristics^17,18^, confounding the results. Alternatively, researchers have developed a zebrafish manipulation strategy (i.e., vertebrate automated screening technology, VAST) that enables automatic sample rotation and screening of zebrafish larvae, which are confined in a thin-walled round glass capillary^14^. Although it presents a powerful platform suitable for most zebrafish studies, VAST involves contact-based sample confinement, which may alter some zebrafish morphological properties and cause samples with large size variations to become stuck when screening morphological abnormities^19,20^. In this regard, there is an unmet need to develop contactless immobilization and manipulation strategies in which external force fields are used to provide strong confinement and orientation control while minimizing external physical contact. Moreover, current zebrafish phenotyping methods usually require the subjective evaluation of features via the visual inspection of 2D image stacks instead of an automated quantitative analysis based on consistent criteria. This adversely affects reproducibility between studies, especially when large populations need to be morphologically characterized.

Classic acoustofluidic techniques, which combine acoustic manipulation and fluid mechanics, have been reported as a contactless approach for translational and rotational manipulation of small objects (e.g., nanoparticles, cells, and *C. elegans*)^21–30^. When trying to improve and stabilize rotational manipulation via acoustofluidics, acoustic streaming with a single vortex pattern is an ideal candidate^31–37^; a single vortex pattern provides a more stable and consistent fluid manipulation pathway than multiple streaming patterns involving complex interactions. Although the non-contact nature and liquid environment make acoustofluidic techniques attractive, scaling up the single vortex pattern for use with larger organisms (such as zebrafish larvae in millimeter scale) has proven challenging based on the existing design schemes of acoustofluidic devices. For most existing acoustofluidic-based rotational manipulation devices, the size of the single vortex is limited to 0.5–2 times the acoustic wavelength depending on the mechanism used^22,38,39^. As a result, targeting the generation of a single vortex streaming pattern ~1 mm in width for use with zebrafish would require an acoustic frequency below ~1 MHz to be applied. Considering that the velocity of acoustic streaming decreases as the frequency decreases^39–44^, single vortex streaming generated by these low-frequency signals based on existing acoustofluidic device setup will be insufficient to rotate large organisms that are in the millimeter scale.

**Fig. 1:**
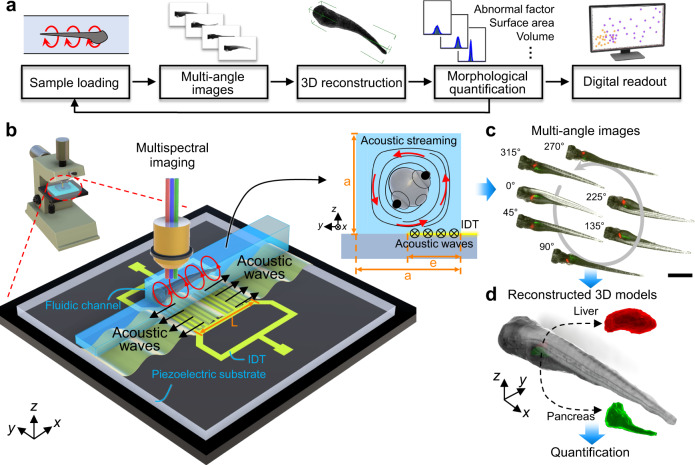
Schematic of the ART system with high-speed 3D multispectral imaging for contactless morphological phenotyping of zebrafish larvae. **a** Flow chart of the working mechanism of the ART system. **b** Illustration of the experimental configuration of the acoustofluidic chip for rotational manipulation of zebrafish larvae mounted on a conventional optical microscope platform. The chip consists of an IDT fabricated on a LiNbO_3_ piezoelectric substrate which generates acoustic waves and a patterned fluidic channel aligned parallel to the lateral side of the IDT (*y* axis) with half of its width on the IDT. The zebrafish larvae in the channel can be rotated by polarized acoustic streaming in a single vortex pattern in the *y*–*z* planes, which was induced by acoustic waves propagating in the ±x direction. The three key parameters contributing to the features of the vortex tube are denoted by “a” for the width of the square cross-section of the channel, “e” for the width of the effective IDT area, and “L” for the length of the IDT. **c** Multiple labeled organs of the larvae can be imaged using the corresponding fluorescent wavelength during rotation. Scale bar: 1 mm. **d** From this multi-angle sequence of microscope images, 3D models of different internal organs of interest can be reconstructed, assembled, and quantified as digital readouts using a computer-vision-based algorithm for subsequent quantitative phenotypic analysis of morphological characteristics.

Herein, we demonstrate an acoustofluidic rotational tweezing (ART) system that enables high-speed morphological phenotyping of zebrafish larvae in a contactless manner (Fig. 1a). Leveraging the designed polarized single streaming vortex excited by acoustic waves, contact-free rotational manipulation and quantitative analysis of zebrafish larvae are conducted. We show that a wide size range of zebrafish larvae with different morphologies can be rapidly (i.e., ~1 s/fish) and stably rotated in our device without changing their morphological characteristics. Both zebrafish bodies and internal organs can be visualized through multispectral imaging. Then, 3D model reconstruction^45–47^ is applied and quantified as digital readouts that enables 3D morphology study (e.g., volume, surface area, and abnormality factor) in an automated manner based on a simple sequence of rotating 2D image captures. In this process, the whole animal or its organs are seen as large particles whose properties can be quantified. By handling a group of zebrafish samples via *a* cytometry-like workflow as shown in Fig. 1a, the ART system can provide digital readouts for a group of samples and further conduct statistical analysis on morphological characteristics of interest for zebrafish phenotyping research, in a similar manner that the conventional flow cytometry processes with cell samples. This ART technology is compatible with a conventional optical microscope and can be applied at various imaging resolutions for different specific applications. We validate our technology by phenotyping the effects of acute ethyl alcohol exposure on body morphology and liver size in 5 days post fertilization (dpf) zebrafish larvae. The information gathered using our technology can provide objective and consistent criteria to improve the reliability of the large-scale phenotyping results. These features enable quantitative comparisons of measured data, thus overcoming the drawbacks associated with traditional, manual analysis of zebrafish phenotypes, which can be subjective, labor-intensive, and lack repeatability between different labs. We believe that the ART with 3D imaging, which combines a contactless acoustofluidic manipulation technique, a conventional optical microscope, and a computer-vision-based processing pipeline, will benefit various biomedical and biological applications, including accelerating drug discovery and simplifying testing of personalized therapies in animal models.

## Results

### Working mechanism of the ART

By rotating the zebrafish larvae with single vortex acoustic streaming, the ART can capture multiple 2D images from different angles (Fig. 1). These multi-angle photos are used to reconstruct a 3D model of the fish for subsequent quantitative phenotypic analysis of morphological features. Here, a computer-vision-based algorithm is used to reconstruct the 2D images into a 3D model of the larvae (Fig. 1c, d) for further phenotypic analysis. The acoustofluidic chip used for rotational manipulation of zebrafish larvae consists of an interdigitated transducer (IDT) fabricated on a lithium niobate (LiNbO_3_) piezoelectric substrate which generates acoustic waves (Fig. 1b). A patterned fluidic channel with a square cross-section is aligned parallel to the lateral side of the IDT (*y* axis) with half of its width on the IDT (Fig. 1b, see Supplementary Fig. 1 for detailed device characterization with definitions of the geometric variables). By applying an AC voltage signal to the IDT, surface acoustic waves (SAWs) are generated that travel along the length of the channel symmetrically (in the ±x direction along the surface of the substrate) with respect to the center of the IDT. The SAWs obliquely leak into the liquid and induce a body force that generates acoustic streaming^25,48–50^. Due to the confined nature of the channel, the fluid above the IDT is jetted upwards by this body force, where it subsequently recirculates along the top of the channel before traveling down the opposite wall and forms a single unidirectional vortex pattern on the *y–z* planes (Fig. 1b). Thus, while the SAWs propagate in the ±x directions, the resulting functional acoustic streaming pattern is a single vortex that lies on the *y–z* planes with its center near the middle of the channel. We characterize this as polarized acoustic streaming.

**Fig. 2:**
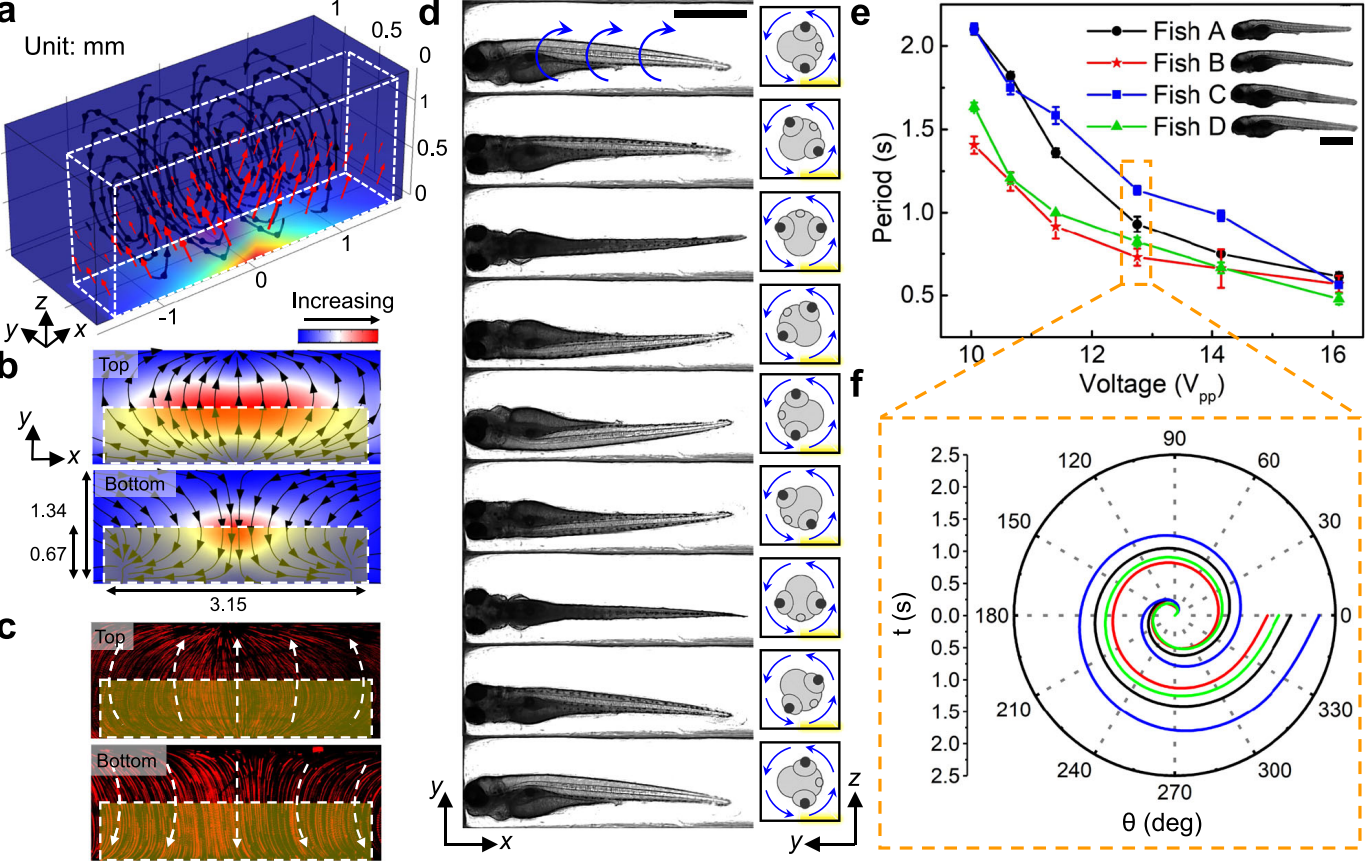
Working mechanism and the performance of the acoustofluidic chip for high-speed, rotational imaging of 5 dpf zebrafish larvae. **a** Results from numerical simulation show that the body force (red arrows) generated by leaky SAWs can induce a vortex streaming pattern within the IDT area. The body force is applied to the liquid above the IDT which is highlighted by the white dashed box. The color of the channel indicates the amplitude of the body force. **b** Numerical and **c** experimental demonstration of acoustic streaming in the zebrafish rotation area on the xy-planes close to the channel top and bottom, respectively. The yellow shaded box indicates the IDT area. The experimentally measured streaming pattern (**c**) represented by the trajectories of 1-μm diameter fluorescent particles, as visualized in a composite of stacked images, matches with the numerically calculated streaming pattern (**b**) driven by acoustic leaky waves generated by the IDTs. **d** Image sequence showing a cycle of the rotational motion of a 5 dpf anesthetized zebrafish larva in the acoustofluidic device. The larva was stably rotated counterclockwise with respect to the yz-plane by the fluid-induced drag force. Scale bar: 1 mm. **e** The rotation periods of four typical 5 dpf zebrafish larvae (length: 3.43–3.6 mm, width: 0.63–0.71 mm) as a function of the driving voltage (V_pp_). The rotational periods vary among larvae since each larva has unique characteristics for body shape, size, and density distribution. Overall, the rotational speed of the zebrafish larvae increases as the driving voltage increases. Data are graphed as the mean ± SD (*n* *=* 6). Scale bar: 1 mm. **f** The rotation angle over a single rotational cycle with respect to time for four typical zebrafish larvae at 12.75 V_pp_. Source data is available as a source data file.

We developed a 3D numerical model to investigate the mechanism of the polarized single vortex acoustic streaming and optimized the device design for stable rotational manipulation (see “Methods” and Supplementary Note for detailed device design and numerical investigation). Notably, through our numerical investigation, we found that a single vortex pattern with its center aligned in the middle of the channel cross-section can be generated for any size channel, as long as the “e = 0.5a” condition is satisfied, and the vortex pattern extends along the channel for the distance of the IDT length (L) (e: width of the effective IDT area, a: width of the channel; see Supplementary Fig. 1 for schematic of geometric definitions). In other words, following the “e = 0.5a” rule, the width and length of the vortex tube can be adjusted by tuning the channel width (a) and the IDT length (L), respectively. As a proof of this result, we numerically and experimentally validated the formation of single vortex streaming within two different channels with a square cross-section (see Supplementary Fig. 3 for the results of the channel with a = 0.8 mm and Fig. 2a–c for the results of the channel with a = 1.34 mm). Our results imply that larger or smaller channels could be designed to handle various organisms based on their sizes, and the single vortex acoustic streaming tube can be scaled accordingly to enable the acoustofluidic rotational manipulation of different model organisms (e.g., *C. elegans*, shrimp, *Xenopus* embryo, or mouse embryo).

### Acoustofluidic-based rotational manipulation of zebrafish larvae

Having numerically and experimentally verified the structure of the acoustic vortex streaming patterns in the channel, we now leverage this contactless acoustofluidic manipulation technique to rotate 5 dpf zebrafish larvae. This will facilitate rapid microscopic imaging of the organism from multiple angles. When acoustic excitation was applied to generate the vortex streaming in the surrounding liquid, a larva was trapped at the center of the channel, stably rotated by the fluid-induced drag force, and then observed from multiple viewing angles (Fig. 2d, Supplementary Video 1). The formation of a single vortex with a zebrafish larva in the channel was confirmed as shown in Supplementary Fig. 4. Due to the complex nature of the fluid motion within the channel, variations in the zebrafish’s feature sizes (e.g., body shape, size, and mass distribution) may slightly affect the rotation period. This variance in the resulting rotation velocity depends on the streaming velocity at which the fluid motion reaches a stable state when interacting with the zebrafish. However, it is important to note that, while the rotation period may vary for different sized zebrafish, a large enough drag force can quickly bring the zebrafish into a stable and consistent rotation mode with a constant angular velocity. This is achieved by applying a high input voltage to generate vortex streaming. Here, the rotation period was characterized using four randomly selected 5 dpf larvae. By adjusting the peak-to-peak voltage (V_pp_) of the AC signals from 10 V to 16 V, the period for a 360° rotation decreased from ~2 s to ~0.5 s (Fig. 2e, f, Supplementary Video 2).

The optimized input voltage was set at 12.75 V_pp_ for future manipulations. This voltage level yields rapid and stable rotational manipulation since the drag force generated by the fluid streaming is sufficiently large to overcome the inertia of the zebrafish. In order to confirm that the ART platform can provide rotational manipulation without changing the innate morphological characteristics of the sample, we compared the shape of the zebrafish at equilibrium states before rotation, during rotation by streaming activated at 12.75-V_pp_ voltage, and after rotation back to its equilibrium position. This comparison was performed for three 5 dpf zebrafish larvae without malformation, with mild malformation, and with severe malformation in shape, and no obvious differences were observed due to the presence of the single vortex streaming (Supplementary Fig. 5). Although a higher voltage would be preferable since it can induce a faster rotation speed, a tradeoff was made to match the rotational speed and the camera frame rate in order to obtain a clear image stack. Also, an input voltage under 20 V_pp_ can commonly be generated by a single function generator without an amplifier, which keeps the whole system more compact. Although primarily performed on 5 dpf zebrafish larvae, the ART device is also compatible with the manipulation of zebrafish at different stages of development, which have different morphologies (e.g., 4 dpf larva as shown in Supplementary Fig. 7 and 0 dpf embryo as shown in Supplementary Fig. 8). One interesting finding from our experiments is that when a zebrafish embryo (0 dpf, prior to tail extension) is loaded into the device, vortex streaming can be generated in the perivitelline fluid within the chorion and drive the rotation of the yolk. This observation has an implication for acoustofluidic-enabled, non-contact rotation, wherein the internal structures of the embryo (i.e., the yolk) can be manipulated without rupturing the external membrane (Supplementary Fig. 8) so that the need for an additional dechorionation step can be eliminated. Furthermore, many protocols for chemical/drug screening studies require the existence of the chorion, as well as testing of a large number of embryo samples for improving statistical confidence in the results^51–53^.

### 3D multispectral phenotyping of zebrafish larvae using the ART

**Fig. 3:**
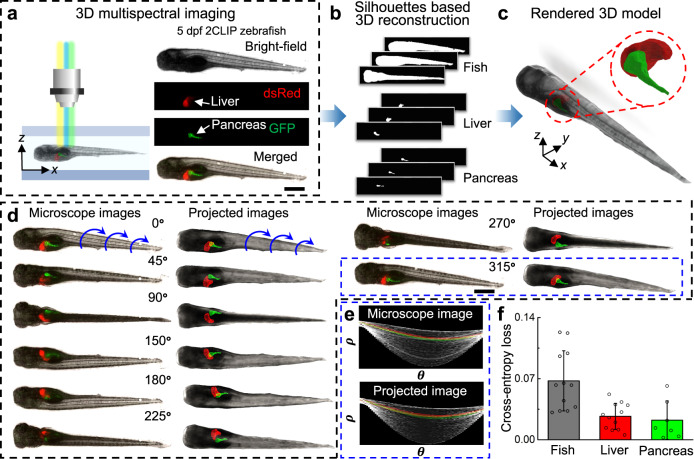
3D multispectral imaging and phenotyping of zebrafish larvae by ART. **a** A 5 dpf 2-CLIP zebrafish larva is exposed to a bright-field and corresponding fluorescent illumination during acoustofluidic rotation for multi-view, multispectral observation of the body, liver, and pancreas, respectively. The composite optical image of the bright-field, DsRed, and GFP images of the same larva shows the relative positions of the pancreas and liver within the zebrafish in a lateral view. Scale bar: 500 µm. **b** The silhouette-based, multi-viewpoint, 3D reconstruction for the zebrafish body, liver, and pancreas at different rotational angles, respectively. **c** The reconstructed and rendered 3D model which includes the zebrafish body, liver, and pancreas. **d** Comparison between the optical image sequence and projected 3D model images at the corresponding viewing angles (see Supplementary Video 3 for more detailed comparison). Scale bar: 500 µm. Representative image set of the projected images is from five independent reconstruction calculations (*n* *=* 5). **e** Comparison between the Hough transform plots of the features of the microscope image and the projected image at 315° in (**d**). No obvious difference is detected between the two Hough transform plots, which means that the reconstructed 3D model is consistent with the optical images of the real zebrafish larva. **f** The binary cross-entropy loss calculated by comparing microscopic images and the corresponding re-projected images of the 3D reconstructed point clouds from different viewpoints for the fish body, liver, and pancreas. The cross-entropy loss calculates the divergence from the viewpoints with respect to the classification accuracy. *n* *=* 12 for fish body and liver, *n* *=* 7 for pancreas. Data are graphed as the mean ± SD. Source data is available as a source data file.

As demonstrated in the previous section, when the acoustic waves are turned on, a zebrafish larva inside the channel is trapped and uniformly rotated. Thus, images of zebrafish larvae at different viewing orientations can be captured under a conventional microscope. By using a bright-field light source, the external morphology of the larvae can be visualized and reconstructed. Expanding on this capability, if the larvae have been treated to express fluorescent proteins or tagged with fluorescent markers, internal systems (organs, nerves, etc.) can be highlighted for visualization in vivo from different viewing angles. That is, by using an appropriate fluorescent light source, internal organs can be specifically targeted, imaged, and analyzed within the digitally-reconstructed 3D model of the fish. To demonstrate this capability, the 2CLIP zebrafish line (*Tg(fabp10:DsRed; ela3l:GFP)*^*gz12*^)^54,55^ was used for multi-angle bright-field and fluorescent imaging within the ART device. These larvae have been engineered to express red and green fluorescent proteins in their liver and pancreas, respectively. These zebrafish larvae can be imaged under the bright field, DsRed, and GFP fluorescent wavelengths for visualization of the entire body, liver, and pancreas, respectively (Fig. 3a). With the ART with 3D multispectral imaging, multiple 2D axial-view images of the zebrafish and its organs are captured. To obtain comprehensive quantitative data that enables 3D morphology study, the shape-from-silhouette 3D reconstruction method^47,56^ was customized to reconstruct these multispectral 2D images to 3D models with the body and internal organ structure (Fig. 3a–c, see “Methods” for a detailed processing pipeline). The dimensions of the pixel volume used for zebrafish body and organ reconstruction were set as 20 × 20 × 20 μm^3^ and 5 × 5 × 5 μm^3^, respectively. When the cross-section dimensions of the zebrafish is gradually shrinking along the body, selecting a larger pixel size may induce a roughness artifact to the zebrafish tail. This could be further optimized by using a smaller pixel volume or variable pixel dimensions along the body. Here, a tradeoff was made between the reconstruction speed and model precision in order to rapidly reconstruct the zebrafish with sufficient accuracy in a continuous mode.

For validation, we compared the projected images of the reconstructed 3D model with multispectral microscope images at the corresponding viewing orientations (Fig. 3d, Supplementary Video 3) and their Hough transform plots (Fig. 3e, see “Methods” for details) which can quantify the major features in the 2D images by plotting their length (*ρ*) and corresponding angles (*θ*) and is commonly used in computer vision to quickly find differences in similar objects. Good correspondence between the projected images and microscope images was found via visual comparison of the similar images of the two groups and their similar Hough transform plots. To validate the accuracy of the 3D model more comprehensively, the binary cross-entropy loss was calculated to evaluate the variance of the 3D model from all viewpoints (Fig. 3f, see “Methods” for detailed analysis). Specifically, the cross-entropy loss of one viewpoint is calculated by identifying if the re-projected 2D points on a viewpoint are also present in the original 2D silhouette of the corresponding microscope images (a “0” loss value for 2D points present in the original silhouette and a “1” loss value for not present). This validation indicated that the re-projections from our 3D reconstructed model had a >90% level of fitting compared to the original images. Thus, the 3D model can not only accurately provide the 3D shape and the relative position between the body and the internal structures of interest, but also reliably quantify the morphological characteristics (e.g., volume, surface area, and profile). In addition, we performed the rotation, imaging, and 3D reconstruction of the zebrafish embryo to validate the reliability of our 3D reconstruction method (Supplementary Fig. 9). Owing to its relatively regular geometry, the volume (V) and surface area (SA) of a yolk prior to tail extension can be estimated as a sphere with V = 1.87 × 10^8^ μm^3^ and SA = 1.59 × 10^5^ μm^2^, respectively. By the aforementioned algorithm, the reconstruction yielded similar values (V = 1.86 × 10^8^ μm^3^ and SA = 1.57 × 10^5^ μm^2^) to the spherical estimate. As an additional validation, we conducted a study in which five 3D liver models of a 5 dpf larva was reconstructed from the imaging record of five different rotation cycles using our algorithm. The images of the reconstructed 3D models, when analyzed from the same angular perspective, yielded similar contours and size estimates, with volume and surface area mean standard deviations of 6.5003 ± 0.0618 (×10^5^ µm^3^) and 1.5803 ± 0.1286 (×10^4^ µm^2^), respectively (Supplementary Fig. 10). The results of these validation studies support the conclusion that the ART with 3D-imaging can be precise and accurate enough to provide qualitative and quantitative 3D data for zebrafish phenotyping research.

### Quantification of ethanol-induced morphological abnormalities

**Fig. 4:**
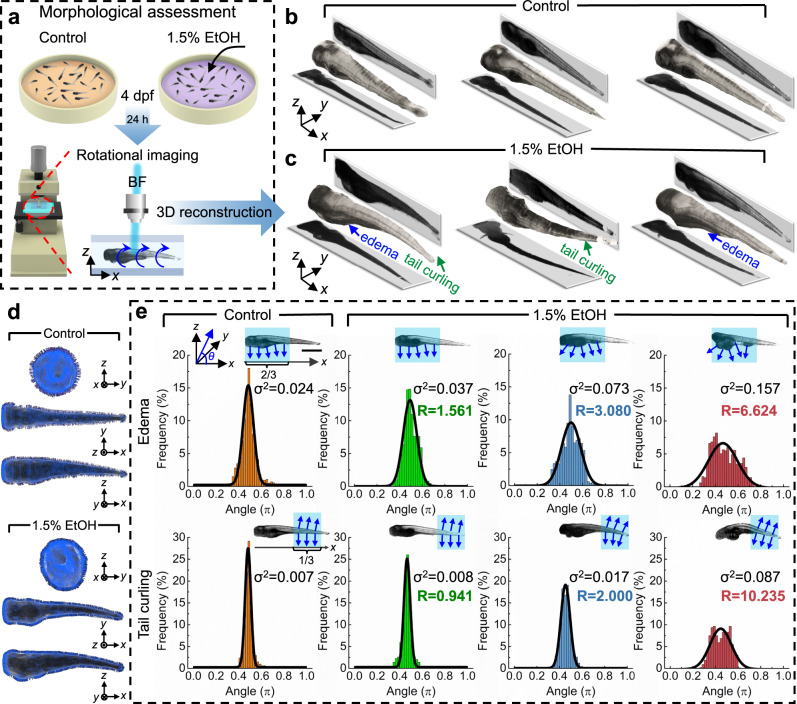
Phenotyping of 5 dpf zebrafish larvae after acute ethanol (EtOH) exposure (1.5% for 24 h) using the ART. **a** Zebrafish larvae were raised in egg water containing 1.5% EtOH at 4 dpf and cultured for 24 h. The control group was raised without EtOH exposure for comparison. Both groups of larvae were imaged and digitally reconstructed at 5 dpf to compare and evaluate the morphological abnormalities induced by EtOH. **b**, **c** Qualitative 3D morphological assessment of zebrafish larvae by comparing the 3D reconstructed models of three zebrafish larvae from the control group (**b**) and from the 1.5%-EtOH group (**c**), respectively. The reconstructed 3D models show that acute EtOH exposure can induce morphological abnormalities in the zebrafish body, which includes edema and tail curling. **d** Distribution of normal vectors on the surface of reconstructed 3D models of a zebrafish larva from the control group and 1.5%-EtOH group, respectively. **e** Distributions of the angle (*θ*) between normal vectors and the *x*-axis for 3D models of a larva of control group and three larvae of 1.5%-EtOH group with increasing degrees of edema and tail curling. The angles of the anterior two thirds and posterior one third of the larva are calculated to quantify the level of edema and tail curling, respectively. With larger deformations, the angles have a wider distribution based on the histogram and Gaussian fitting curve, as reflected on the value of the variance (σ^2^). The “abnormality factor” ($R = \sigma ^2/\sigma _0^2$) shows the degree of morphological abnormalities by calculating the ratio of variances in the larvae of the 1.5%-EtOH group (σ^2^) and the averaged variance of the control group ($\sigma _{0E}^2 = 0.0237 \pm 0.0091$ and $\sigma _{0T}^2 = 0.0085 \pm 0.0032$; *n* = 15). Scale bar: 1 mm. Source data is available as a source data file.

Zebrafish have frequently been used as model organisms for studying the consequences of acute alcohol abuse, which may induce hepatomegaly and morphological abnormalities. To assess the capabilities of the ART for 3D imaging and quantitatively measuring objects with irregular shapes, we next studied abnormalities resulting from EtOH exposure in zebrafish. For these experiments, 4 dpf zebrafish larvae were raised in egg water containing 1.5% EtOH and cultured for 24 h (Fig. 4a). A control group was raised without EtOH for comparison. Both groups of larvae were rotationally imaged using the ART at 5 dpf. 3D models of the larva body were reconstructed based on the multi-view images to evaluate the morphological abnormalities resulting from acute EtOH exposure. The larvae in the control group show straight tails and consistent body sizes, as seen in Fig. 4b and Supplementary Video 4. In comparison, sample 3D models in Fig. 4c show the commonly observed morphological abnormalities in the EtOH group, namely generalized edema and tail curling. It is worth noting that for various biological studies, higher resolution imaging would be vital as it could provide information about the cellular mechanics for detailed research in drug discovery, cancer therapy testing, and environmental toxicity studies. For example, signs of edema in zebrafish larvae would usually be a result of the combined effect of cellular changes that can be divided further into two sub-groups: pericardial edema (PE) and yolk sac edema (YSE)^57,58^. While both cases correspond to the abnormal shape for different parts of the zebrafish, the cells and intercellular spaces involved have increased in volume due to the accumulation of water. As shown in Supplementary Figs. 11 and 12, by using the ART with a higher resolution imaging system, we can observe the edema with cellular level changes from different viewpoints. Moreover, benefiting from digital examination of the resulting 3D model, not only can the morphology information be obtained from 2D images in the sagittal plane (*x–z*) and the coronal plane (*x–y*), the shape and dimensions in the transverse plane (*y–z*) can also be detected as shown in Supplementary Fig. 13.

**Fig. 5:**
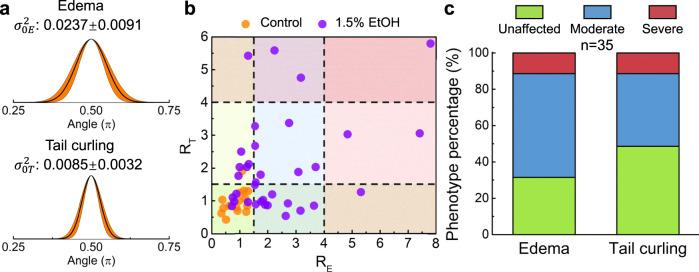
Morphological phenotyping based on automated quantitative measurements. **a** Gaussian fitting curves of the angle distribution statistics of 15 larvae in the control group. The averaged variances of the angle distribution for edema and tail evaluation are $\sigma _{0E}^2 = 0.0237 \pm 0.0091$ and $\sigma _{0T}^2 = 0.0085 \pm 0.0032$, respectively. **b** Statistical distribution of the “abnormality factors” ($R = \sigma ^2/\sigma _0^2$) for edema and tail curling in the control group (*n* = 15) and the 1.5%-EtOH group (*n* = 35). Based on statistical analysis, the larvae within the range of *R* *≤* 1.5, 1.5 *<* *R* *≤* 4, and *R* *>* 4 are categorized as unaffected, moderate, and severe, respectively, for morphological abnormality level. **c** The percentage of phenotype classification (as unaffected, moderate, or severe) of the morphological abnormalities after the 1.5% EtOH exposure. Source data is available as a source data file.

Although purely visual comparisons between the experimental groups can offer some insights, quantitative metrics can provide consistent criteria for a more objective statistical analysis and deliver more reliable phenotyping results. Since the edema (including both PE and YSE)^57,58^ and tail curling are inherently reflected on the shape of the zebrafish, we developed an analytical method to provide a quantitative parameter, based on the reconstructed 3D model, as a criterion for evaluating the degree of the morphological defects. We first calculated the normal vectors on the surface of zebrafish larvae based on the 3D model (Fig. 4d). We then calculated the angle (*θ*) between each normal vector and the *x-*axis and plotted histograms of the angle distribution with Gaussian fits (Fig. 4e). In this way, the shape characteristics of the larval body can be embodied by the Gaussian fitting curve and quantitatively reflected on the value of curve variance (σ^2^). As the edema and tail curling are mainly associated with the anterior two thirds and posterior one third of the body, respectively, the distribution of the angles within the corresponding section reflects the degree of defects. As shown in Fig. 4e, zebrafish larva with a larger degree of edema and tail curling have a wider angle distribution as compared to the angle distribution for the control group. This is quantitatively reflected by the variance (σ^2^) values. Specifically, zebrafish edema usually refers to the swelling of the fluid accumulation near the yolk (located around the anterior two thirds of the zebrafish), which will generate normal vectors with a wider angle distribution (Supplementary Fig. 14). Hence, a larger variance in the distribution of the normal vector angles can be quantitatively associated with a higher level of edema. Similarly, tail curling will produce a larger variance in the normal vector angles within the posterior one third of the body depending on the degree of curling (Supplementary Fig. 14). Thus, this analytical method based on the reconstructed 3D models can be used as a baseline standard to quantify the degree of abnormalities. Here, we can define a factor for quantifying the morphological impact of EtOH exposure called the “abnormality factor”, which we define as ${R} = {\upsigma}^2/{\upsigma}_0^2$, where σ^2^ and ${\upsigma}_0^2$ are the variance in the angle distribution of the target zebrafish in the experimental group over the averaged variance of zebrafish of the control group (${\upsigma}_{0{E}}^2 = 0.0237 \pm 0.0091$ for edema and ${\upsigma}_{0{T}}^2 = 0.0085 \pm 0.0032$ for tail curling; *n* = 15; Fig. 5a), respectively. Based on the value of the abnormality factor, EtOH-induced morphological abnormalities can be categorized into six groups corresponding to the morphological traits (i.e., edema and tail curling) and levels of abnormalities (i.e., unaffected, moderate, and severe) measured in the imaged fish. Figure 5b shows the statistical distribution of the “abnormality factors” for edema and tail curling of the control group (*n* = 15) and the 1.5%-EtOH group (*n* = 35). Taking the value of *R* as a quantitative reference standard, we can classify the degree of edema and tail curling to three levels, i.e., unaffected (*R* ≤ 1.5), moderate (1.5 < *R* ≤ 4), or severe (*R* > 4). Using these levels, we calculated the phenotype percentage with these morphological abnormalities after the 1.5% EtOH exposure (Fig. 5c). This quantitative evaluation of morphological abnormality provides a reference and quantification standard that is connected to traditional empirical criteria. While edema or tail curling may be induced by different mechanism (e.g., body curling may be induced by shrinkage on one side or expansion on the other or by over or undergrowth of tissue), the statistical study of these abnormalities will aid in the identification of the single or multiple experimental variables which can induce this effect. Furthermore, a retrospective analysis of the resulting 3D model database can quickly locate specific abnormal zebrafish to perform further detailed physiological measurements (e.g., higher resolution imaging on certain parts of the zebrafish). Using our ART platform, we were also able to determine the percentage of the phenotype’s frequency via the aforementioned automated quantification process (see Supplementary Fig. 15 for the entire process flow). Our results showed that edema is a more common morphological abnormality, as induced by acute EtOH exposure, when compared to the frequency of tail curling, with ~70% of the larvae displaying edema at the belly after EtOH exposure, and ~10% being classified as a severe deformation. In comparison, ~50% of the EtOH-exposed population displayed tail curling, of which roughly 10% were considered severe. These findings are consistent with recent published research which has shown a strong correlation between edema and EtOH exposure. This has drawn parallels to the impact on human infants suffering from fetal alcohol syndrome^59,60^.

### Quantitative analysis of the impact of EtOH on zebrafish liver size

**Fig. 6:**
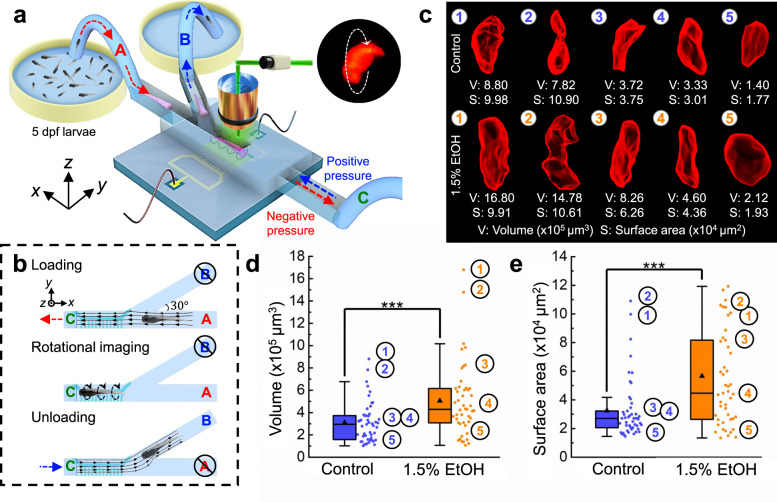
Statistical analysis of zebrafish liver size in response to EtOH exposure. **a** The schematic shows the setup for imaging zebrafish samples via the semi-automated flow control system of the ART platform. A Y-shape channel enables the successive steps of high-throughput sample loading, rotational imaging, and sample unloading. **b** Schematic illustrating the channel flow settings for sample loading, rotational imaging, and the unloading processes. Branch C has a negative pressure applied and outlet B is blocked when zebrafish larvae are introduced from inlet A. No pressure is provided to branch C during the rotational imaging process. After rotational imaging, the pressure at branch C is switched to positive and the inlet A is blocked to eject the zebrafish larva through outlet B. The process is repeated continuously until all the larvae have been imaged. **c** Typical reconstructed 3D models and quantification of five zebrafish livers from the control group and the 1.5%-EtOH group, respectively (see Supplementary Video 6 for 3D models). **d**, **e** Statistics of the liver volume distribution and the surface area distribution of the control group and 1.5%-EtOH group, respectively. Based on the statistical analysis, the liver size of the 1.5%-EtOH group is more likely to be larger than that of the control group. This suggests that acute EtOH exposure can induce hepatomegaly in zebrafish larvae. *n* = 49 and 47 for control and 1.5%-EtOH groups, respectively. (One-way ANOVA, ***P *<* 0.0005, *P* = 0.000461 for volume and *P* = 0.000316 for surface area). All boxplots indicate median (center line), mean (triangle), 25th and 75th percentiles (bounds of box), and ±1.5 × IQR (interquartile range) (whiskers). Source data is available as a source data file.

In addition to the external morphological abnormalities, acute EtOH exposure has also been correlated with abnormalities in the zebrafish liver. This is similar to the negative impact that alcohol abuse can have on liver function in humans, presenting itself as steatosis, or “alcoholic fatty liver disease”^61–63^. Here, we demonstrate a step towards automated 3D reconstruction of zebrafish larvae using the ART. As shown in Fig. 6a, a Y-shaped channel with 3 branches was designed for efficient loading, rotational imaging, and unloading of batches of zebrafish larvae. Briefly, branch “C” was connected to a syringe pump for controlling the flow direction to load or unload the sample from inlet “A” and outlet “B”, respectively. The branches “A” and “B” were mounted to a three-way pinch valve (one normally open, one normally closed) so that the flow in one branch of either “A” and “B” was blocked when the sample passed through the other branch. Figure 6b and Supplementary Table 1 show the status of the pump and the single three-way valve in the pre-programmed modes of sample loading, imaging, and unloading. In the sample loading mode, the valve was set to A-open/B-closed while the pump was withdrawing fluid and sample from inlet A. The tubing at inlet A was manually directed towards a zebrafish at the beginning of the sample loading process to make this channel loading more efficient. When the sample reached the imaging area, the pump was stopped so that the sample can be imaged. After the sample was imaged, the three-way valve was switched to A-closed/B-open mode where the pump then dispensed the fluid and sample through outlet B to a container with fresh DI water. The switching between the operating modes of the flow system was done manually, and the IDT was kept active during the entire process. Using this semi-automated ART system, rotational imaging under DsRed fluorescent illumination and 3D reconstruction were successively performed on batches of 5 dpf 2CLIP zebrafish larvae from both the 1.5% EtOH group and the control group to investigate the effect of EtOH on the liver size within the developing larva (Supplementary Video 5). Liver size quantification via volume and surface area measurements were gathered from both groups (*n* = 49 and 47 for the control and the 1.5%-EtOH groups, respectively) and compared by statistical analysis (One-way ANOVA). From the 3D reconstruction, we found that the larvae in the 1.5%-EtOH group typically had livers with larger average sizes and less regular shapes than those in the control group (see Fig. 6c for representative 3D models). The liver volume distribution (Fig. 6d) and liver surface area distribution (Fig. 6e) show that, on average, EtOH exposure caused liver sizes to enlarge 73.7% by surface area (3.26 × 10^5^ µm^3^ for the control group vs. 5.66 × 10^5^ µm^3^ for the EtOH group) and increased 62.7% by volume (3.10 × 10^4^ µm^2^ for the control group vs. 5.07 × 10^4^ µm^2^ for the EtOH group). A shape change between the control group and the EtOH group was also measured (as shown in Supplementary Fig. 16) indicating an increase in the cell volume, which would explain the overall liver volume change. The increase in the liver surface area of the EtOH-exposed group is most likely attributed to an increase in surface roughness and liver shape variance, which increased the surface area faster than the liver volume. By analyzing the 3D model generated using the ART system, we extracted the outline features from three standard planes (i.e., sagittal plane (*x–z*), coronal plane (*x–y*), and transverse plane (*y–z*), as shown in Supplementary Fig. 17). This shows that while the overall liver volume will increase after EtOH treatment, the liver shape has also been changed from a “V” like shape to a rounder shape. This is most possibly due to the increase in cell volume and compression against adjacent tissues (e.g., edema effect in zebrafish belly). Our ART system was thus used to demonstrate that EtOH exposure can induce morphological changes in the liver, such as enlarged size and surface roughness, and this can be measured in a semi-automated manner. Altogether, these results demonstrate the capabilities of ART for conducting high-throughput morphological phenotyping of live vertebrates.

## Discussion

In this work, we have demonstrated an acoustofluidic rotational tweezing (ART) platform that integrates contactless rotational manipulation, multi-view multispectral imaging, and 3D model reconstruction for quantitative phenotypic evaluation and analysis of morphological abnormalities in batches of zebrafish larvae. Specifically, through both numerically modeling and experimental investigation, our acoustofluidic device can generate a polarized acoustic streaming single vortex with controllable parameters. This unique mechanism can create a strong single vortex acoustic streaming tube in millimeter-scale with high frequency (over 20 MHz) and low input voltage (~10 V_pp_).

This feature would be challenging for previous acoustic platforms due to the strong correlation between the vortex dimension and wavelength^49^ and the balance between the frequency and voltage^30,38,64^. Our acoustofluidic technology was successfully demonstrated to stably and rapidly rotate model organisms with different sizes and shapes in a contactless manner. This could potentially benefit not only large-scale morphological evaluations but also enable the long-term, periodic phenotype quantification of model organisms at different stages of development. Although not originally targeting for use with advanced imaging systems, our ART system does contain specific features comparable to the state-of-art 3D phenotyping techniques. For example, optical projection tomography, as one of the most powerful imaging methods for live rodents, can provide label-free structural images for morphometrics in developmental biology. 3D morphology information can be generated in a single imaging session^65,66^. Also, Micro-CT, a commonly used technique for visualization of skeletal tissue or embryonic soft tissues, can provide images with high spatial resolution^65,66^. Each method has advantages and limitations. For example, optical projection tomography will need fixation of the sample during imaging which may potentially make it difficult to preserve certain morphology information^66^. Meanwhile, due to the imaging mechanism, real-time viewing will be difficult to obtain^66,67^. Micro-CT, on the other hand, has a similar potential issue with morphology distortion due to fixation or desiccation as scanning in a liquid medium would be problematic due to the low contrast between tissue and water^68^. Compared to these methods, the advantages of our ART system are the non-contact feature and phenotyping in a near-continuous flow mode that can efficiently image and digitize a large number of specimens. Meanwhile, the overall system is more compact and flexible as different imaging or analysis tool can be integrated. It would also be worth noting that just like optical projection tomography, the imaging quality of our system would depend on the optical imaging lens. Different lenses would be needed to achieve various depth of focus and resolutions. Furthermore, the system could be further developed to enable fully automated processing which serves as a target for our next stage of research.

We performed a proof-of-concept demonstration by phenotyping a large population of 5 dpf zebrafish larvae with respect to changes to body morphology and liver size due to EtOH exposure. Leveraging our acoustofluidic rotational imaging mechanism and recent advances in computer vision techniques, we can reconstruct a 3D model with a composite of bright-field and fluorescent images of the zebrafish and highlighted portions in a rapid and efficient manner. Any uneven rotation angle between each video frame would affect the resulting 3D reconstruction. As the corresponding angles are related to the camera matrix (rotation matrix) that was used to determine the isosurface of the 3D model, any rotational non-uniformity corresponding to an incorrect estimate for the image angle will cause distortions in the 3D model. In order to have a consistent rotation angle in our experiment, the optimal acoustic voltage was used to generate a strong acoustic streaming and drag force. Thus, in this stable rotation mode, the force torque would be zero and since angular momentum is conserved, this effectively results in uniform rotation (see Supplementary Fig. 18 for proof of angle consistency). This 3D reconstruction produced a high-resolution model for the detailed analysis of phenotypic data. While classic 3D scanning and reconstruction techniques (e.g., confocal laser scanning microscopy) suffer from discretization artifacts, our acoustofluidic method takes images from multiple angles and combines the images via 3D reconstruction, hence minimizing optical interference effects resulting from various depth. A complete cycle of loading, rotational imaging under three different light fields, 3D reconstruction of the body and organs, and unloading a zebrafish usually took less than 59.1 s using the ART with 3D multispectral imaging (Supplementary Table 2). During the entire process, each cycle of rotation required ~1 s. Combined with the loading and unloading time, the complete rotational imaging of one specimen required less than 11.2 s for imaging under a single light field and less than 14.4 s for imaging under three different light fields. This rotational imaging speed of the ART platform is comparable to that of the VAST system^14^, whereas the sample loading efficiency is slightly lower. This process could be improved by integrating a robotic arm that draws zebrafish samples from a 96-well plate or adding a propeller inside the reservoir. Combining all the features of our ART system, we are able to quickly examine a large population of specimens and produce digital 3D multispectral models of each specimen for subsequent detailed analysis. Considering the zebrafish larvae or the structures of interest as “large particles”, the workflow of the ART system is similar to that the flow cytometry systems process with cell samples. This workflow dramatically reduces the time required for tasks involving microscope-based multispectral examination of small animal models. Moreover, the statistical data is drawn from quantified consistent criteria for phenotyping studies. This can accelerate drug discovery as well as minimize the number of personnel hours required and subjective interference compared to previous manual specimen loading and observation methods.

Previous studies looking into morphological abnormalities in zebrafish have relied on the subjective evaluation of the abnormality level. With our platform, we can rotate and image the fish from different viewing angles for enhanced observation, and we can directly transfer the complete, multispectral, digital zebrafish model into an automated machine-vision-based analysis program. This technique is performed using only a conventional optical microscope and could potentially standardize the process of morphological phenotyping. Altogether, we believe our ART platform shows great potential as the basis for non-contact live vertebrate manipulation and transport with culturing, 3D imaging, and semi-automated analysis capabilities for applications such as drug discovery, personalized cancer therapy testing, and the evaluation of environmental toxicity effects.

## Methods

### Device design, fabrication, and operation

The IDT is patterned on 128° Y-cut lithium niobate wafer (Precision Micro-Optics, USA) by e-beam evaporation of 5 nm Cr/50 nm Au followed by standard photolithography and etching. As shown in Supplementary Fig. 6, an 8-μm photoresist (SU-8 10, MicroChem Inc., USA) was spin-coated over the IDT area as an electrical insulating layer. Another 100-nm Au layer was deposited on this photoresist layer by e-beam evaporation for sample observation enhancement. The fluid channel is composed with a PDMS channel, square glass capillaries, and silicone tubing. The PDMS channel was fabricated through standard soft-lithography and mold-replica procedure. The channel mold is fabricated by CNC machining. The PDMS channel is designed with a lower height near the IDT aperture to align the larvae in a consistent position within the vortex generation zone. After the PDMS channel was cut to a suitable size with two open ends, it was bonded to the substrate via an oxygen plasma surface treatment. Then square glass capillaries with a 1 × 1-mm^2^ cross section (VitroCom, USA) were attached to the ends of the PDMS channel using silicone glue. Flexible silicone tubing with a 1.59-mm inner diameter (EW-06411-62, Masterflex, USA) were connected with the ends of the square capillaries for sample loading and unloading. The end of the tubing connected to branch C (Fig. 6b) was connected to a 10-ml syringe (302995, BD Medical, USA) mounted to a syringe pump (78-8210, KD Scientific, Inc., USA), and the tubing connected to inlet A and outlet B was mounted to a pinch valve (98302-42, Cole-Parmer, Inc., USA). The motion of the pump and the valve was controlled by a programmable Arduino board (Elegoo, UNO R3). Supplementary Fig. 19 shows a photo of the assembled acoustofluidic chip. External wires were connected to the electrodes of the IDT using silver epoxy (MG Chemicals, USA) to provide the activation signal, which are AC voltage signals generated by a waveform generator (AFG3102C, Tektronics Technology Corporation, PA, USA) and amplified by an amplifier (25A250A, Amplifier Research, USA). For most cases of zebrafish larvae imaging, the AC signals were set to 12.75 V_pp_ in voltage and 23.9 MHz in frequency. This was applied to activate vortex streaming and to rotate the zebrafish larvae.

### Theoretical model and numerical simulation

The acoustic streaming in the channel is governed by the continuity equation and the Navier-Stokes equations^40,41^:

1 $$\rho _0\nabla \cdot {\mathbf{v}}_{{\mathrm{dc}}} = 0$$

2 $$\rho _0\left( {{\mathbf{v}}_{{\mathrm{dc}}} \cdot \nabla } \right){\mathbf{v}}_{{\mathrm{dc}}} = - \nabla p_{dc} + \mu \nabla ^2{\mathbf{v}}_{{\mathrm{dc}}} + \left( {\mu _b + \frac{1}{3}\mu } \right)\nabla \left( {\nabla \cdot {\mathbf{v}}_{{\mathrm{dc}}}} \right) - {\mathbf{F}}$$

where *ρ*_0_ is the fluid density, *μ*_*b*_ is the bulk viscosity, *μ* is the shear viscosity, **v**_dc_ is the steady streaming velocity, and **F** is the body force as a source term to activate the acoustic streaming. For acoustic streaming activated by SAW propagating in the +x direction, **F** decays as it propagates from the center of the IDT, and can be expressed as^40^:

3a $$F_x = - \left( {1 + \alpha _1^2} \right)A_m^2\omega ^2k_ie^{\left[ {2\left( {k_ix + \alpha _1k_iz} \right)} \right]}$$

3b $$F_y = 0$$

3c $$F_z = - \left( {1 + \alpha _1^2} \right)A_m^2\omega ^2k_i\alpha _1e^{\left[ {2\left( {k_ix + \alpha _1k_iz} \right)} \right]}$$

where $$\alpha = i\alpha _1$$.

Numerical simulations were performed using the finite element method (FEM)-based software package, Comsol Multiphysics 5.4 (COMSOL AB, Sweden) based on the aforementioned theoretical model. The COMSOL “Laminar Flow” interface with FEM formulations of the governing equations presented in (1) and (2) was applied to the 3D solution domain. This domain is a section of fluid in the channel above the IDT area (domain length: 3.4 mm, IDT length: 3.15 mm). The body force in equation (3) was applied to the portion of fluid domain which lies above the IDT, as marked by the white dashed frame Fig. 2a because the SAWs and thus the body force in this area is much stronger in this region. An “outlet” condition was applied to the two ends of the channel. And, the boundary conditions were set to “no slip” (${\mathbf{v}}_{{\mathrm{dc}}} = 0$) at the bottom and sides of the channel. The model was solved by a “stationary” solver to get the steady streaming pattern in the fluid domain. The numerical model was used to explore the acoustic streaming patterns generated within the fluid domain for different value of variables, *e.g*., effective IDT area overlapped with the channel bottom (Supplementary Fig. 2) and channel width (Fig. 2 and Supplementary Fig. 3), and provide reference for the optimization of chip design.

### Zebrafish larvae preparation

The zebrafish (*Danio rerio*) transgenic line (*Tg(fabp10:DsRed; ela3l:GFP)*^*gz12*^) which expresses red fluorescent protein in the liver and green fluorescent protein in the pancreas was used in this work^54^. Zebrafish studies were approved by the Institutional Animal Care & Use Committee at Duke University (protocol # A154-18-06). Zebrafish embryos/larvae were cultured in egg water at 28 °C and 14-hour light/10-hour dark conditions. For 3D imaging of the internal organs, skin pigmentation needs to be reduced to obtain optimal observation of the fluorescent signal, as expressed by the internal organs, from multiple viewing angles. In the EtOH exposure experiments in this work, 0.003% 1-phenyl-2-thiourea (PTU, Sigma, USA) was added to the egg water to inhibit melanogenesis and to reduce the zebrafish skin melanization^69^ so that the fluorescent signal expressed by the organs can be observed more clearly. All the experiments in this work were performed on zebrafish up to 5 days post fertilization. The zebrafish larvae were anesthetized with 1× Tricaine (~0.2 mg/ml; Sigma, USA) to prevent active movement during rotational imaging. For EtOH exposure, the zebrafish larvae in the experimental group were moved to egg water containing 1.5% EtOH at 4 dpf and cultured for 24 h. During imaging, the zebrafish larvae were moved to DI water containing 1× Tricaine (Sigma, USA) and 1% Pluronic^®^ F-127 surfactant (Sigma, USA) to keep the zebrafish larvae anaesthetized and to improve the smoothness of the rotational motion. The immersion time of the zebrafish larvae in this solution is less than 3 min. Viability checks by assessing the health of zebrafish larvae were performed on three groups of 50 2CLIP zebrafish larvae (Supplementary Fig. 20a). The three groups were treated as follows at 2 dpf: Group 1 (control group): 1× Tricaine for 5 min; Group 2: 1× Tricaine + 1% Pluronic^®^ F-127 for 5 min; and Group 3: 1× Tricaine + 1% Pluronic^®^ F-127 for 5 min, and rotational imaged using the ART system for 30 s during this immersion time. After the treatment, the larvae were collected in cell strainers, gently rinsed with fresh DI water, and cultured for 48 h. The health of the zebrafish larvae was assessed at their 4 dpf by observing their morphological appearance, survivability, response to touch stimuli, swim bladder appearance, and heartbeat. From their morphological appearance, no abnormalities in body bending were observed in all the groups. As shown in Supplementary Fig. 20, the rate of survival, touch response, the appearance of swim bladder at 4 dpf, and heart rate in the Group 2 and Group 3 matched those of controls. The result of the health assessment shows that the routine use of the ART system does not cause obvious harmful effects to zebrafish larvae.

### Imaging and shape-from-silhouette (SFS) 3D reconstruction

The digital reconstruction procedure is briefly summarized as follows: (1) capture the background image in an empty channel before the zebrafish enters, then inject the zebrafish and record a sequence of images for one rotation cycle corresponding to an angle *θ* from 0 to 2*π*. (2) Perform preliminary image processing by subtracting the background image from the series of rotational images, which digitally and visually isolates the segments of the zebrafish. (3) Binarize the image series to obtain the 2D silhouettes (Fig. 3b). (4) Initialize a 3D grid of voxels according to the 2D silhouette’s boundary and size. (5) Back-project the 2D profiles into the 3D framework from the outside “world perspective” and obtain the matching intersections. (6) From this point, we can generate a watertight 3D visual “hull” of the target object (Fig. 3c). Note that although our experimental configuration is different from the common camera system for applying SFS (a moving object vs a moving camera), the tensor describing the camera frame of reference (i.e., the camera matrix) can be considered identical since the camera center is defined as the world origin. Thus, the relationship between the 2D image and 3D coordinates in the real world can be expressed as: $a_n = K\left[ {R_n|t} \right]A_n$, where *a*_*n*_, *A*_*n*_ are corresponding 2D and 3D coordinates, *K* is the intrinsic matrix of the camera, and $[R_n|t]$ is the extrinsic matrix which can be divided into a rotation matrix *R*_*n*_ and a translation matrix *t*. Here, *n* denotes the *n*^th^ visual angle that relates to the rotation angle of the zebrafish.

The series of images captured using the three illumination fields are recorded sequentially and then angularly aligned to obtain one composite series of images. The angular alignment is achieved by recording one extra image of the fluorescent fields with a slight overexposure, such that the outline of the zebrafish can be identified and matched with the corresponding angular perspective of the respective bright field image. Then, the fluorescent image series are aligned starting from the angle corresponding to the extra matching image. After the fusion of the three illumination fields, the 3D multispectral model is calculated using the same camera matrix, and the size of each voxel of the organs (i.e., liver and pancreas) is set to be 5–10 times smaller than the whole body to achieve a finer reconstruction resolution.

The microscope images and videos are recorded using an upright microscope (BX51WI, Olympus) with a CCD camera (CoolSNAP HQ2, Photometrics), a ×2.5 objective lens (MPLFLN2.5X, Olympus), and the CellSens 1.18 (Olympus Corp., JPN) software. One of the most important parameters in the 3D reconstruction is the determination of the camera matrix which connects the 3D coordinates in the real world with the 2D position in the image. The classic camera matrix can be denoted as: $P = K\left[ {\left. R \right|t} \right]$, *K*_3×3_ is the camera intrinsic matrix that contains the camera configuration itself as:

$$K = \left[ {\begin{array}{*{20}{c}} {f_x} & s & {x_0} \\ 0 & {f_y} & {y_0} \\ 0 & 0 & 1 \end{array}} \right]$$

where $\left( {f_x\,f_y} \right)$ is the focal length in pixels that can be derived as: $f_x = f/dx$ and $f_y = f/dy$, *dx*, *dy* are the length of the single pixel in the image, $\left( {x_0\,y_0} \right)$ is the image center, and *s* is the shear factor of the camera. It can be decomposed into three matrixes as:

$$\left[ {\begin{array}{*{20}{c}} {f_x} & s & {x_0} \\ 0 & {f_y} & {y_0} \\ 0 & 0 & 1 \end{array}} \right] = \left[ {\begin{array}{*{20}{c}} 1 & 0 & {x_0} \\ 0 & 1 & {y_0} \\ 0 & 0 & 1 \end{array}} \right] \times \left[ {\begin{array}{*{20}{c}} {f_x} & 0 & 0 \\ 0 & {f_y} & 0 \\ 0 & 0 & 1 \end{array}} \right] \times \left[ {\begin{array}{*{20}{c}} 1 & {\frac{s}{{f_x}}} & 0 \\ 0 & 1 & 0 \\ 0 & 0 & 1 \end{array}} \right]$$

which correspond to the 2D translation, scaling, and shear, respectively. $[\left. R \right|t]$ serves as the extrinsic matrix including the rotation matrix and the translation matrix, which can be referred to as the camera motion in 3D. The rotation matrix *R* can be decomposed into the rotation components along three axes: $R_{3 \times 3} = R_X \times R_Y \times R_Z$ where, for our case, the rotation angle of the *x*-axis is changing with different frames during rotation. And the 3D translation vector *t* can be parametrized as $t_{3 \times 1} = \left[ {0,\,f\cos \left( \varphi \right),\,f\sin \left( \varphi \right)} \right]^T$ following the general definition.

For a single zebrafish 3D reconstruction, images with the total frame number ranging from 15 to 31 are used. The differences in the total number of frames depending on the individual differences in zebrafish (e.g., each zebrafish has a slightly different rotational velocity). Each 2D image has a resolution of 2048 × 2048 pixels which corresponds to a field of view of ~5.33 × 5.33 mm^2^ for the whole image. The end of one rotation cycle is determined by identifying the frame *i* that has the minimum difference with the first image: $\left. i \right|_{\theta = 2\pi } = {\mathrm{arg}}\,{\mathrm{min}}\left( {\left\| {a_i - a_1} \right\|} \right)$. After the number of the frames *N* in one cycle is determined, the rotation angle can be estimated by $\theta _i = 2\pi (i - 1)/N$ as the acoustofluidic device generates a stable rotation with a constant angular velocity for each zebrafish. A photomask is designed with a checkerboard pattern to obtain an accurate camera intrinsic matrix. By taking multiple images of the photomask at different orientations, the camera intrinsic matrix can be calibrated using the camera calibrator toolbox in MATLAB R2016b (MathWorks, USA). Once the camera model parameters are identified, a representation comprised of 3D volumetric voxels can be reconstructed from the 2D binary axial-view images by intersecting all the voxels whose 2D projection is covered by the silhouettes: $V = \mathop {\bigcap}\nolimits_{i = 1:N} {v_i}$, $v_i = \left\{ {\left. {A(x,y,z)} \right|S_i(a_i^\prime ) \, \ne \, 0;a_i^\prime = P_iA_i^\prime } \right\}$, where $A_i^\prime$ is the 3D coordinates of the initial voxels, $a_i^\prime$ is the 2D projection of the initial voxels in *i*th viewpoint, _*Pi*_ is the camera matrix in the *i*th viewpoint, *S*_*i*_ denotes the approximation function determining whether the 2D projection of the voxels is within the silhouettes image. Then the marching cubes algorithm is used to generate a “watertight” surface with a dense point cloud and triangulated meshes from the reconstructed 3D voxel sets. Moreover, for the batch reconstruction process of the image flow model, optimization of the camera matrix is conducted by detecting and updating the translation vector *t* and the region of interest (ROI) since the zebrafish may rotate in a position that has slightly shifted from the center of the image. For this case, a frame of the ROI is defined as a square region with a minimum side length that can cover the segments in the image series and the center of the ROI serves as the center of the zebrafish or of its organs. MeshLab 2016.12^70^ and SolidWorks 2016 (SolidWorks Corp., USA) are used for 3D model texture mapping, rendering and visualization. The 3D reconstruction is implemented using Matlab programming on a desktop with an Intel i7 CPU and 64GB RAM. The runtime for the program to reconstruct a 3D model depends on the total number of image frames. It ranges from 7.97 s to 10.40 s for a zebrafish body reconstruction and 12.44 s to 17.34 s for an organ, excluding the time to digitally save the.STL file (3D model). The data were visualized as curves, histograms, and scatter plots by OriginLab 2018 (OriginLab Corp., USA).

### Validation of the 3D reconstruction

The Hough transform highlights the features of the 2D images by plotting the angle (*θ*) and norms (*ρ*) of the small features in the image in the *x*- and *y*-axis, respectively. The correspondence of two 2D images can be evaluated quantitatively by comparing their Hough transforms. The accuracy of the 3D reconstruction was quantitatively validated by comparing the Hough transforms of the merged microscope optical images with the 2D projected images of the 3D model at corresponding viewing orientations.

The binary cross-entropy loss was calculated to further quantify the accuracy of the reconstructed 3D model more comprehensively. First, the 3D point clouds of the 3D model were re-projected back to the 2D planes from different viewpoints to get the re-projected 2D points. The cross-entropy loss of one viewpoint is calculated by identifying if the re-projected 2D points on a viewpoint are also present in the original 2D silhouette of the corresponding microscope images: ${\cal{L}}_i = 1/N\mathop {\sum}\nolimits_1^N {\ell _{0 - 1}(p,\hat p)}$, where *N* is the number of the re-projected 2D points; *p* and .. are 2D points in silhouette and the re-projected image, respectively; and $\ell _{0 - 1}(p,\hat p)$ indicates whether the loss function ($\ell$) is either 0 or 1 (the loss function is equal to 0 if the re-projected point is also found in the silhouette image, and not present for a value of 1). Then, the total binary cross-entropy loss of the whole 3D model is calculated as the average from all viewpoints: $L = 1/fn\mathop {\sum}\nolimits_1^{fn} {{\cal{L}}_i}$, where *fn* is the number of frames/viewpoints.

### Reporting summary

Further information on research design is available in the Nature Research Reporting Summary linked to this article.

## Supplementary information

Supplementary Information

Description of Additional Supplementary Files

Supplementary Video 1

Supplementary Video 2

Supplementary Video 3

Supplementary Video 4

Supplementary Video 5

Supplementary Video 6

Supplementary Video 7

Reporting Summary

## Data Availability

The datasets supporting the findings in this study are available from the corresponding author upon reasonable request. Source data are provided with this paper.

## Source data

Source Data

## Code availability

The codes used for 3D reconstruction in this study are publicly available at the GitHub repository: 10.5281/zenodo.4422134^71^.
