# Supplementary material for: Acoustofluidic rotational tweezing enables high-speed contactless morphological phenotyping of zebrafish larvae
Source: Nat Commun. 2021 Feb 18;12:1118. doi: 10.1038/s41467-021-21373-3 (PMC7892888; doi:10.1038/s41467-021-21373-3)
Supplement: Supplementary file 1 — Supplementary Information [file 41467_2021_21373_MOESM1_ESM.pdf]

Supplementary Information

Acoustofluidic Rotational Tweezing Enables High-Speed Contactless Morphological Phenotyping of Zebrafish Larvae

Chuyi Chen[1], Yuyang Gu[1], Julien Philippe[2], Peiran Zhang[1], Hunter Bachman [1], Jinxin Zhang[1], John Mai[3], Joseph Rufo[1], John F. Rawls[4], Erica E. Davis[2,5,6], Nicholas Katsanis[2,5,6] and Tony Jun Huang[1*]

[1]Department of Mechanical Engineering and Material Science, Duke University, NC 27708, USA.
[2]Center for Human Disease Modeling, Duke University Medical Center, Durham, NC 27701, USA.
[3]Alfred E. Mann Institute for Biomedical Engineering, University of Southern California. Los Angeles, CA 90089, USA.
[4]Department of Molecular Genetics and Microbiology, Duke University, Durham, NC, 27710, USA.
[5]Advanced Center for Translational and Genetic Medicine (ACT-GeM), Stanley Manne Children's Research Institute, Ann & Robert H. Lurie Children's Hospital of Chicago, Chicago, IL, 60611, USA
[6]Department of Pediatrics, Feinberg School of Medicine, Northwestern University, Chicago, IL 60611, USA

Correspondence and requests for materials should be addressed to T.J.H. (email:tony.huang@duke.edu)

Supplementary Figures

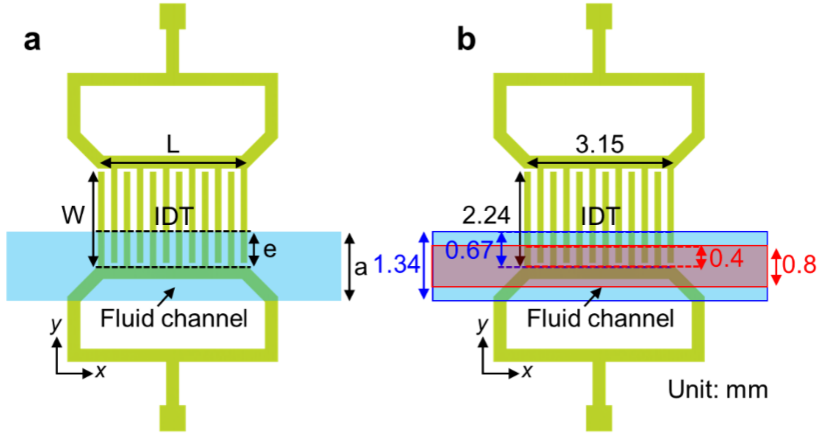

Supplementary Figure 1 | Labeled schematic and geometric variables of the acoustofluidic rotational manipulation device. **a**, Definition of geometric variables relative to device design. The lateral side length and aperture width of the IDT are denoted by "L" and "W", respectively. The width of the square fluid channel is denoted by "a". The fluid channel is aligned parallel to one flank of the IDT. The dashed lines indicate the section of the IDT covered by the fluid channel, which is effective for the generation of acoustic streaming. The width of this effective IDT area is denoted by "e" in this work. The setting of e=0.5a, which indicates placing half of the width of the fluid channel upon the IDT, was applied to generate "polarized" single vortex acoustic streaming; in this configuration, the center of the cross section of the channel overlaps the IDT for non-contact zebrafish rotational manipulation. **b**, Values of the key variables explored in this work. In this work, the geometry of the IDT was designed with L=3.15 mm and W=2.24 mm, containing 21 pairs of IDT fingers with 37.5 µm in width and 2.24 mm in length. This IDT was activated by 23.9-MHz AC signals. The two designs of channels assembled with this IDT following the e=0.5a rule rotation were a=1.34 mm and a=0.8 mm as marked by the blue and red shaded rectangles.

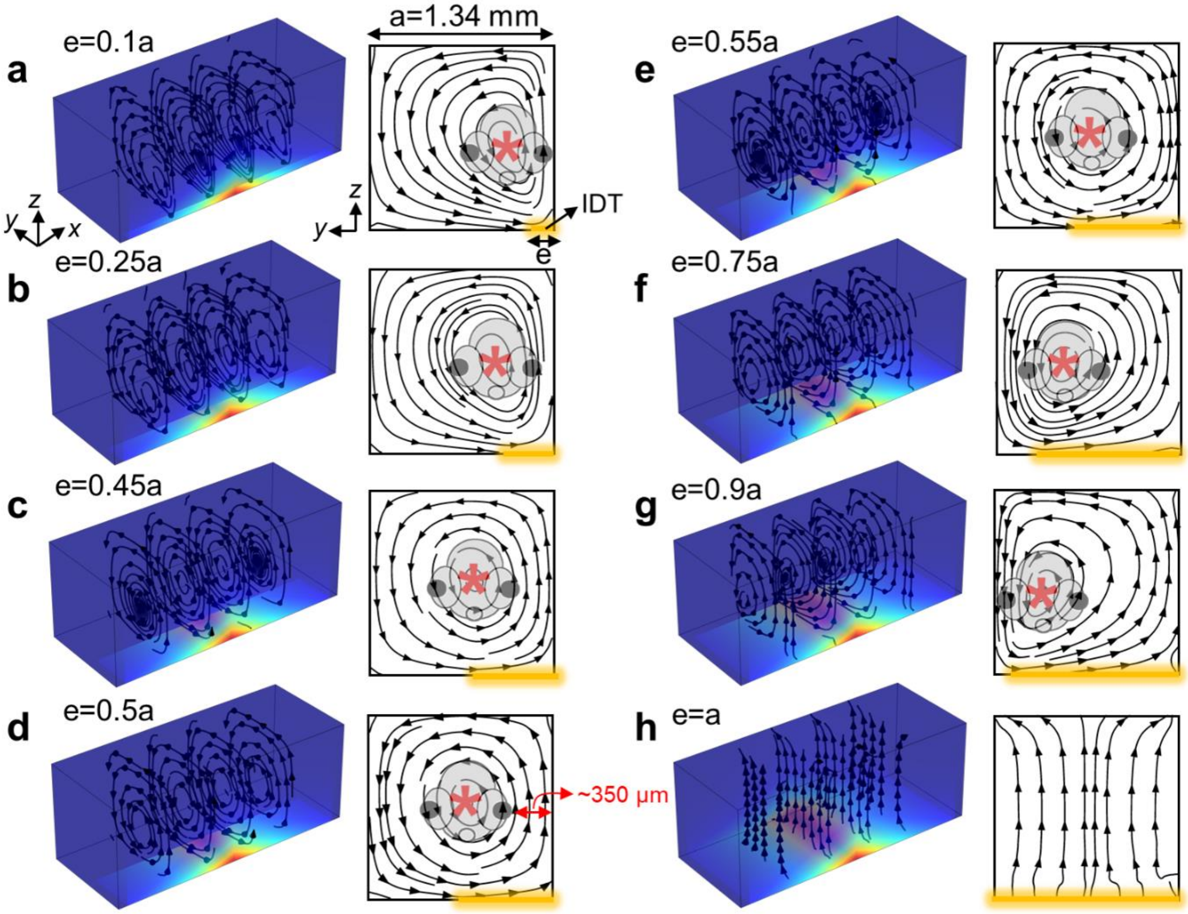

Supplementary Figure 2 | Simulation results showing the effect of width of the effective IDT area (e) to the streaming pattern. Numerical simulations were performed to explore the streaming pattern in the fluid channel with respect to the value of e for the case of a=1.34 mm. **a-h**, Streaming pattern in 3D field and a streaming pattern on the yz-plane at the middle of the simulation domain for the corresponding value of e, respectively. The color shows the amplitude of the body force with red for large values and blue for small values. The red asterisks indicate the centers of the vortex streaming fields, which predict the location of the rotation axis. The zebrafish schematics indicate the predicted positions of stable rotation of a larva with maximum width of 600-µm, a typical value 5 dpf larvae, in the corresponding cases. **a**,**b**,**f**,**g**, When much less or much more than a half of the channel is placed on the IDT, the center of the single vortex is far away from the center of the channel in the y axis. In these cases, the rotation of the zebrafish larvae was predicted to be unstable because of the asymmetric streaming pattern and the possible resistance from the channel wall. **c-e**, If about half of the channel is placed upon the IDT (0.45a≤e≤0.55a), the center of the vortex streaming closely overlaps with the center of the channel in the y axis, where we predict the stable rotation axis of the zebrafish larvae will be. In this range, the distance of the 600-µm larvae to the channel wall will be about 350 µm which allows the zebrafish larvae which fall within the size range (<~850 µm for 5 dpf larvae) to be rotated with a low chance of contacting the channel wall. Thus, the device fabrication of the acoustofluidic zebrafish larvae rotation device was designed to be e=0.5a, which means a half of the channel width was located upon the IDT. **h**, Vortex streaming pattern will not be formed if the whole channel is placed on the IDT.

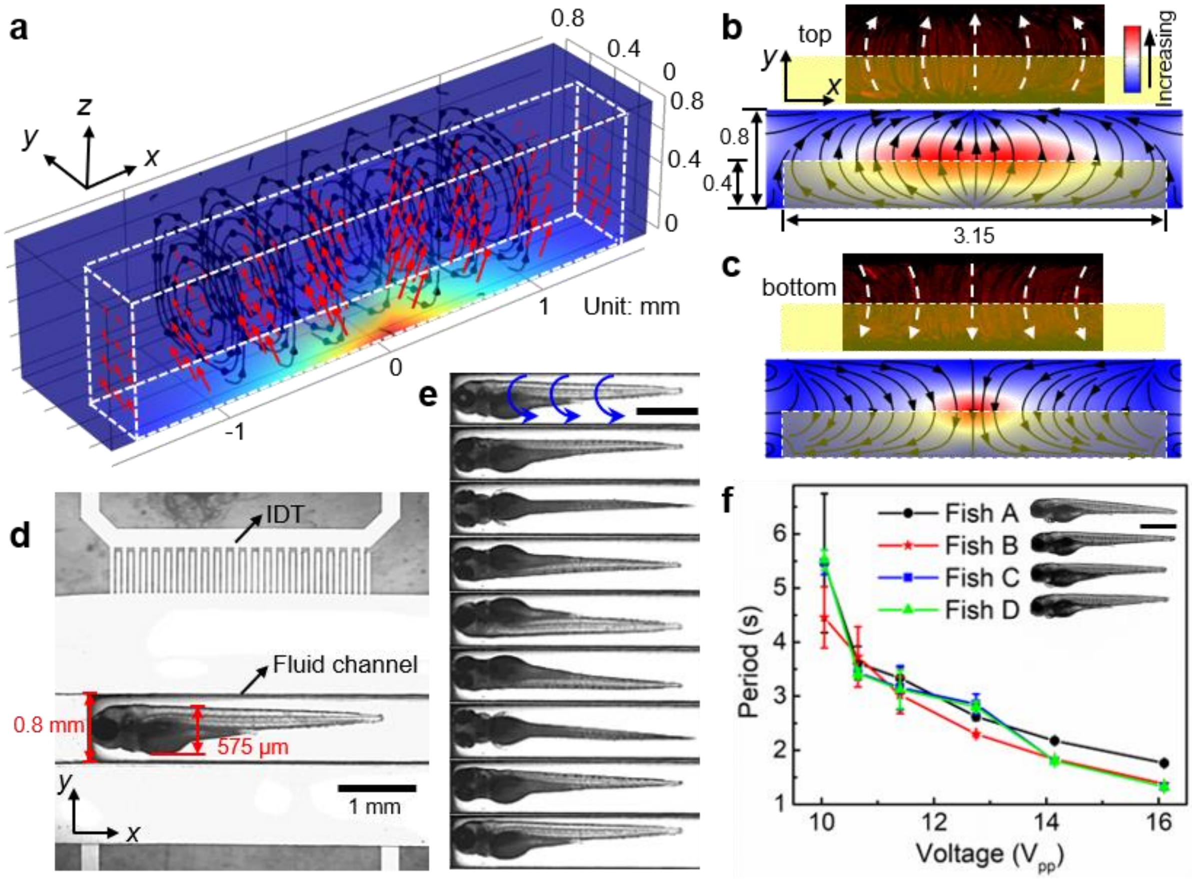

Supplementary Figure 3 | Streaming pattern and zebrafish larvae rotational motion evaluation in a channel with 0.8×0.8 mm^2 in the cross section. a, The distribution of the body force (red arrows) generated by leaky SAWs results in acoustic streaming following a single vortex pattern in the channel with a cross section of 0.8×0.8 mm^2. The body force was applied to the domain upon the IDT area marked by the white dashed frame, which covers half of the channel in width. The color shows the amplitude of the body force with red for large values and blue for small values. Single vortex acoustic streaming pattern on the yz-planes extends along the channel in the x direction and enables the acoustofluidic rotational manipulation of objects that lie in this area. **b**,**c**, Experimental and numerical demonstration of acoustic streaming in the zebrafish rotation area on the xy-planes close to the channel top (**b**) and bottom (**c**), respectively. The yellow shadowing box indicates the IDT area with a length of 3.15-mm and width of 0.4-mm which covers a half of the channel in width. The experimentally measured streaming patterns represented by stacked images of 1-µm particles trajectories match with the simulated streaming patterns driven by acoustic leaky waves generated by the IDTs. **d**, Microscope image shows a 5 dpf zebrafish larva in the device and the relative position of the IDT and fluid channel. **e**, Image sequence showing a cycle of the rotational motion of a 5 dpf zebrafish larva in the microfluidic channel. Scale bar: 1 mm. **f**, Plot of rotational periods against driving voltage (V_{pp}) of four 5 dpf zebrafish larvae by acoustic streaming in a 0.8-mm-channel. The rotation period of four zebrafish larvae with size varying from 3.38 to 3.60 mm in length and 0.60 to 0.63 mm in width were characterized. The rotation speed of the zebrafish larvae in the 0.8-mm channel was approximately 65% slower than that in the 1.34-mm-channel. Scale bar: 1 mm. n=5 for Fish A at 10.05 V_{pp}; n=6 for other points. Data are graphed as the mean ± SD.

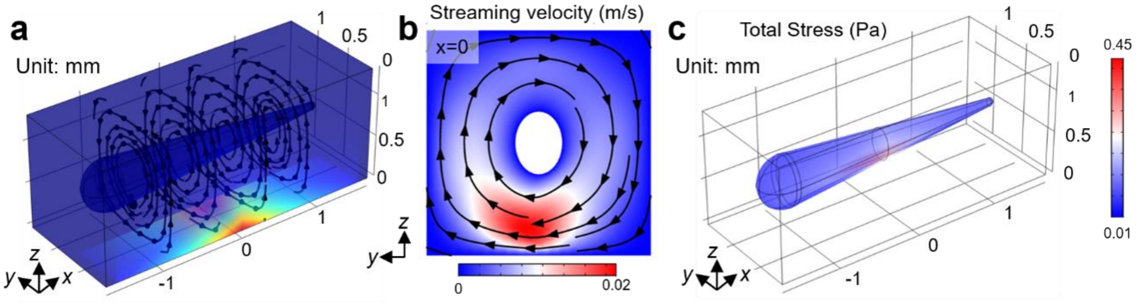

Supplementary Figure 4 | Numerical Simulation results showing the streaming pattern with a zebrafish larva in the channel (a=1.34 mm). A numerical simulation was performed to show the stresses from a single vortex streaming pattern on a larva loaded into the channel. The geometry of a typical 5 dpf zebrafish larva was modeled as an elongated teardrop-shaped cone with a length of 3 mm and a maximum width of 600 μm. The larva was assumed not to rotate with the flow to simplify the simulation. Thus, the boundary condition on the surface of the zebrafish larva was set to "no slip". **a**, Streaming pattern in 3D fluid domain can be formed with a zebrafish larva in the channel. **b**, The resulting streaming velocity and streaming pattern on a 2D cross-sectional plane at x=0. The streamlines with arrows indicate the pattern of acoustic streaming. **c**, The total stress $([-p\mathbf{I} + \mu(\nabla\mathbf{v}_{\text{fluid}} + (\nabla\mathbf{v}_{\text{fluid}})^T)] \cdot \mathbf{n})$ distribution on the surface of zebrafish larva is less than the hydrostatic pressure.

24

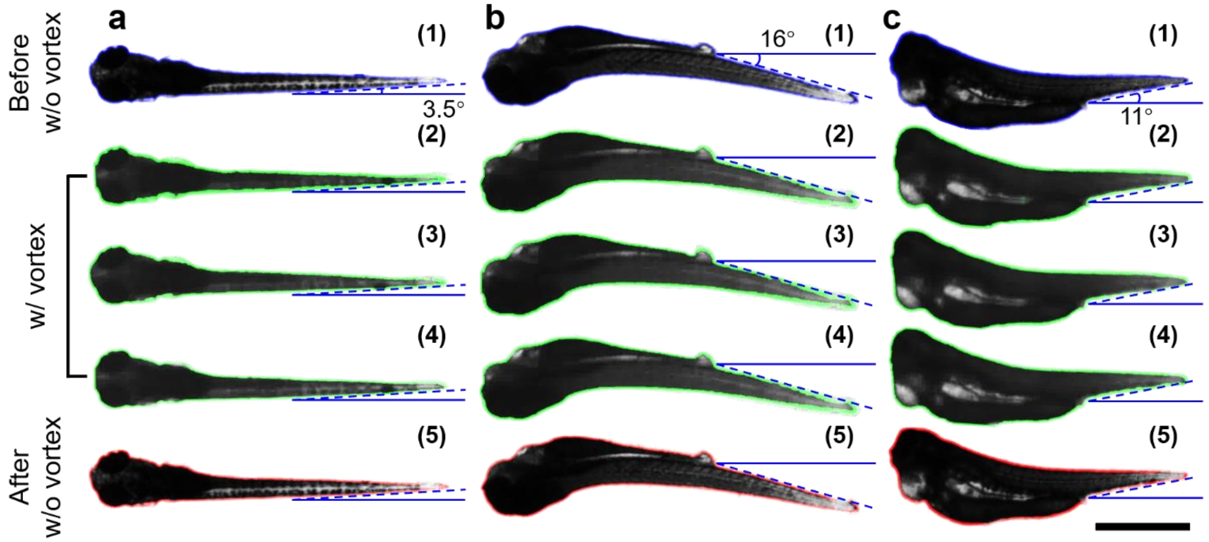

Supplementary Figure 5 | Comparison of zebrafish tail curling before(no vortex streaming), during (with vortex streaming), and after rotation (no vortex streaming). a, A 5 dpf zebrafish larva without edema or tail curling. **b**, A 5 dpf zebrafish larva exposed to 1.5% EtOH shows moderate edema and tail curling. **c**, A 5 dpf zebrafish larva exposed to 1.5% EtOH shows severe edema and tail curling. Scale bar: 1 mm. Figures (1) show the equilibrium status of the zebrafish outside of the vortex, figures (2)-(4) show the zebrafish during different rotation cycles at same corresponding viewing angles as figure (1) with respect to each group, and figures (5) were taken after the vortex vanished and the zebrafish reached equilibrium again. The angle gauges of figures (1) which indicate the original tail curling degree of each group were duplicated to other figures in the corresponding group to compare the angles of tail curling. By comparing angles of the tail curvature before, during, and after rotation by vortex streaming, no change in the degree of tail curling or body shape was detected.

25

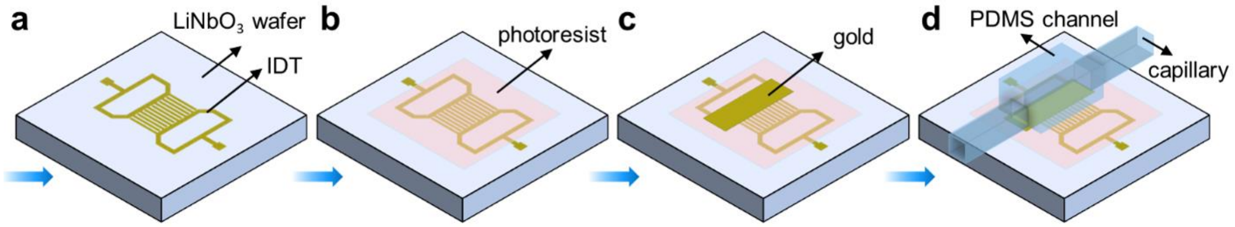

Supplementary Figure 6 | Fabrication steps for the acoustofluidic rotation device for manipulating zebrafish. **a**, An interdigitated transducer is fabricated by e-beam evaporation followed by photo photolithography. **b**, A 8-μm photoresist layer (SU-8 10) is spin coated on the IDT area to electrically isolate the piezoelectric substrate from the zebrafish. **c**, A 100-nm gold layer is deposited upon the photoresist layer to hide the IDT fingers during imaging. **d**, A PDMS channel, fabricated by soft-lithography, is oxygen plasma bonded to the coated substrate and two capillaries with 1x1 mm^2 square cross section are connected to the PDMS channel using a silicone adhesive.

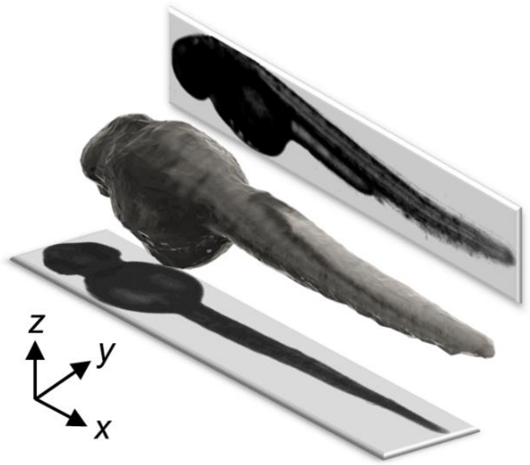

Supplementary Figure 7 | Imaging and 3D reconstruction of a 4 dpf zebrafish larva.

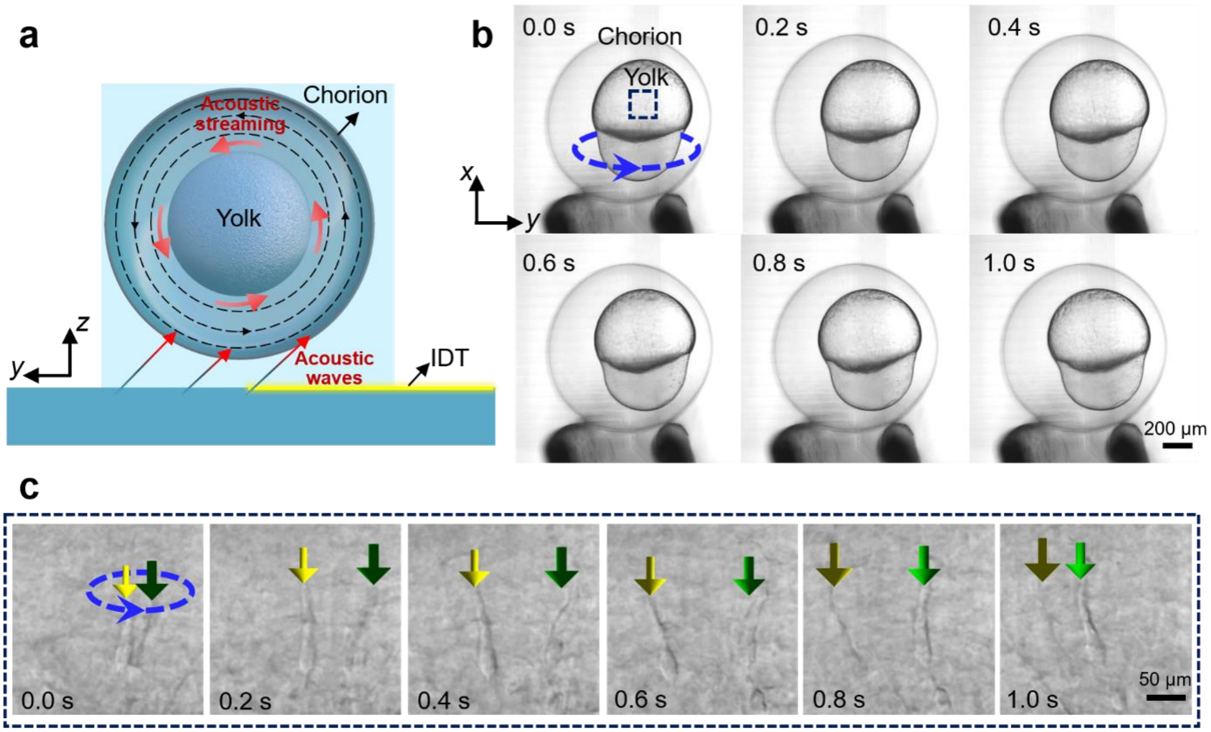

Supplementary Figure 8 | Rotation and imaging of a zebrafish embryo (0 dpf, prior to tail extension). a, Schematic showing the rotation of the yolk in the chorion achieved by leveraging acoustic streaming within the perivitelline fluid of the embryo. The acoustic leaky waves are transmitted through the chorion and generate acoustic vortex streaming in the perivitelline fluid within the chorion which rotates the yolk. **b**, A sequence of images capturing the rotation of the yolk of a ~0.2 hpf zebrafish embryo caused by the acoustic streaming in the chorion. **c**, Magnified images of the region within the black-dashed frame in (**b**), at each respective time step. The yellow and green arrows indicate and follow the movement of the end of two lines of the texture of the developing organism within the yolk, respectively. Five independent embryo rotation experiments (n=5) were used for rotation analysis. Representative embryo images from different angles were chosen to indicate the rotation motion. Scale bar: (**b**): 200 μm, (**c**): 50 μm.

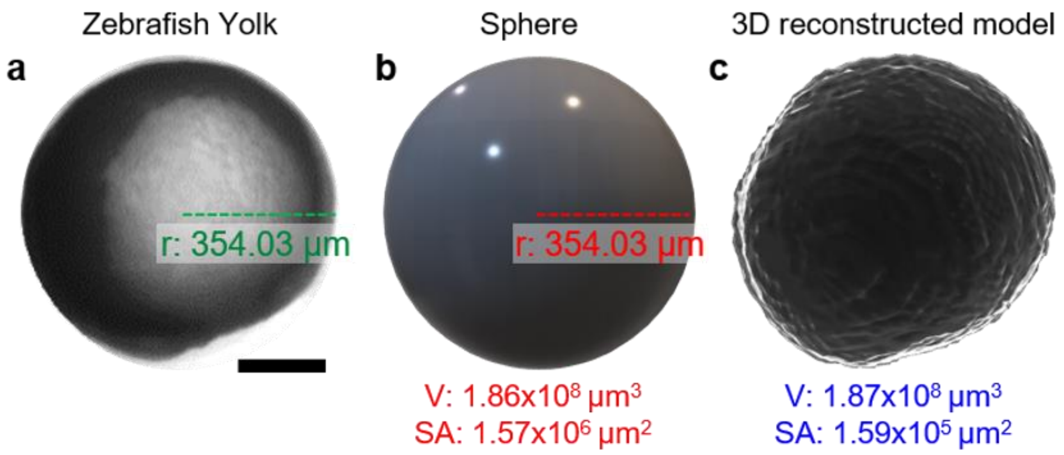

Supplementary Figure 9 | Validation of the 3D reconstruction. a, An image of a quasi-spherical yolk with a radius of 354.03-μm is rotationally imaged and reconstructed. The actual zebrafish yolk size can be modelled as a sphere with a radius of 354.03-μm. Five independent embryo rotation experiments (n=5) were used for rotation analysis. Representative embryo image was chosen to indicate the dimension and compare with the reconstruction results. (**b)** whose volume and surface area were calculated to be 1.86×10^8 μm^3 and 1.57×10^6 μm^2, respectively. **c**, The 3D reconstructed model of the yolk in **a**. The volume and surface area of the 3D reconstructed model was evaluated to be1.87×10^8 μm^3 and 1.59×10^5 μm^2, respectively, which are close to the estimated values computed from the shape of the sphere in **b**. Representative image set of the projected images is from three independent reconstruction calculation (n=3). Scale bar: 200 μm.

31

Cycle #	1	2	3	4	5	Average	STD
**3D model**	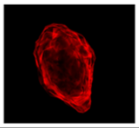	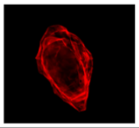	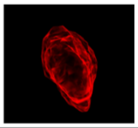	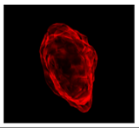	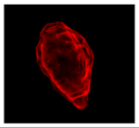		
**Volume** ($\times10^5$ μm^3)	6.5176	6.5791	6.5188	6.4126	6.4734	6.5003	0.0618
**Surface area** ($\times10^4$ μm^2)	1.7813	1.4482	1.5031	1.6183	1.5508	1.5803	0.1286

Supplementary Figure 10 | Validation of the 3D reconstruction based on a liver image at different rotation cycles. The volume and surface area of the same zebrafish liver were evaluated based on five 3D models reconstructed from five different cycles of rotational imaging. The small STD shows that the reconstructed model is consistent between different rotation cycles and the method is reliable.

32

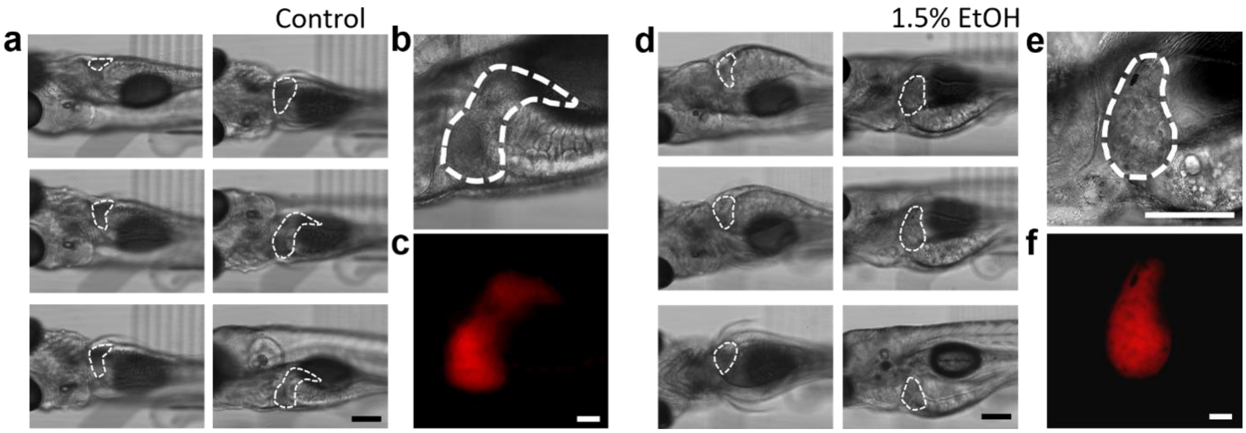

Supplementary Figure 11 | Rotational imaging of the liver of the zebrafish larva for the control group (a-c) and 1.5% EtOH group (d-f). a, Higher resolution imaging of the zebrafish liver at different viewing angles with the liver outline marked by white dash lines. **b**, Magnified liver image. **c**, Fluorescent image of the liver corresponding to (**b**). **d**, Higher resolution images of the ethanol-treated liver at different viewing angles. **e**, Magnified image of the liver. **f**, Corresponding fluorescent image of the liver. Three independent zebrafish rotation experiments (n=3) were conducted in each group for zoomed-in visualization. Representative zebrafish images from different angles were chosen to indicate the rotation motion. Scale bar: (**a**),(**d**): 200 μm, zoomed-in images (**b**),(**c**),(**e**),(**f**): 50 μm.

33

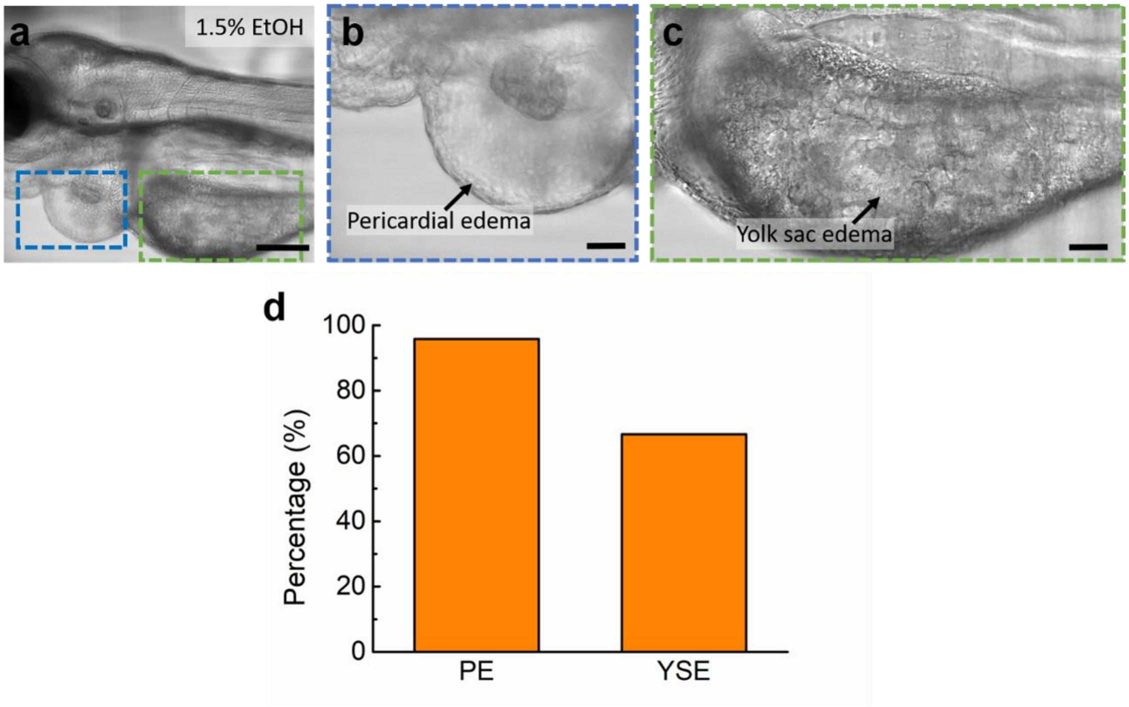

Supplementary Figure 12 | Higher resolution images showing the abdominal shape and cells in the zebrafish larva with ethanol exposure. a, 1.5% EtOH-exposure induced pericardial edema (PE) and yolk sac edema (YSE) in the zebrafish larva. Using higher magnification images, increases in the cell volume and the morphological deformation can be detected using our system for the PE (**b**) and YSE (**c**). Scale bar: (**a**): 200 μm, (**b**) and (**c**): 50 μm. **d**, The percentage of pericardial edema (PE) and yolk sac edema (YSE) in the affected sample in the 1.5% EtOH group in Fig. 5, n=24.

34

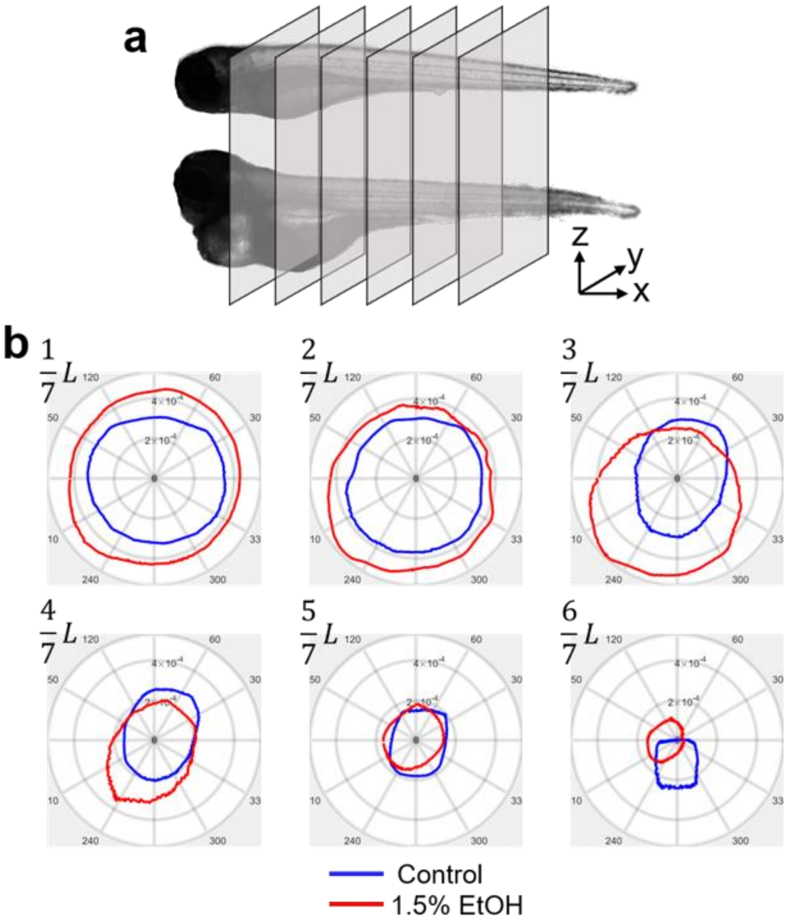

Supplementary Figure 13 | An outline analysis in the transverse (y-z) plane for size differences along x-axis. a, Illustrative images comparing the zebrafish larva with (bottom) and without (top) EtOH treatment. **b,** Comparisons of zebrafish outlines in the transverse plane showing the morphology difference along the zebrafish larva body.

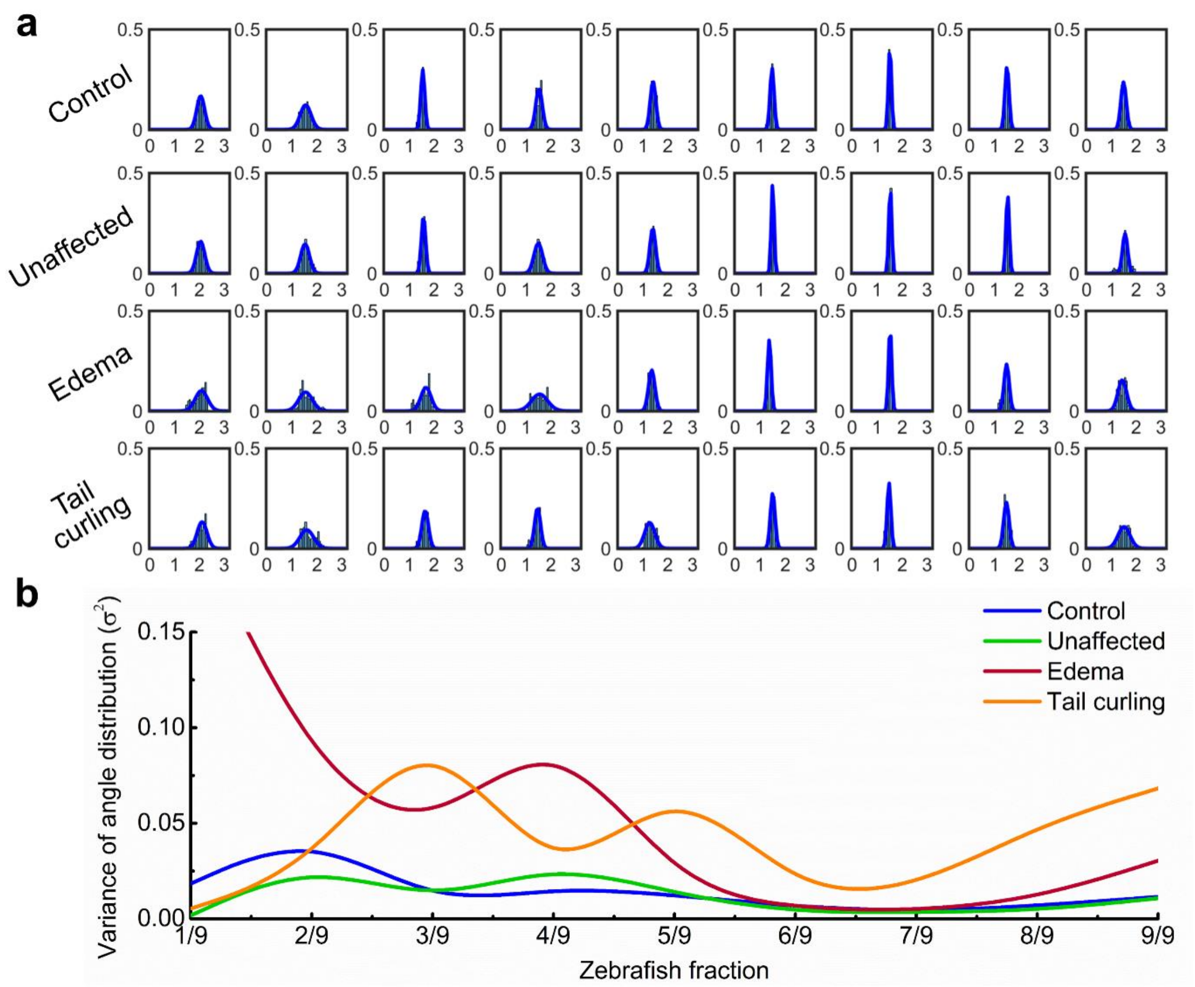

Supplementary Figure 14 | The angle distribution and variance of normal vectors relative to the x-axis along the zebrafish body. a, The angle distribution for four different abnormal categories (i.e., control, unaffected, edema, and tail curling). The zebrafish body is divided into 9 parts along the x-axis and normal vector angles are characterized for each part. **b,** The variance of the angle distribution along the body indicates the relationship between the abnormal level and σ^2. Specifically, the edema is shown to mainly contribute to larger variances in the first two thirds of the body, and tail curling is mainly related to the last one third of the body. Since the edema abnormality appears in almost all EtOH treated zebrafish the variance also shows slightly larger variance in the first two thirds of zebrafish for the tail curling model.

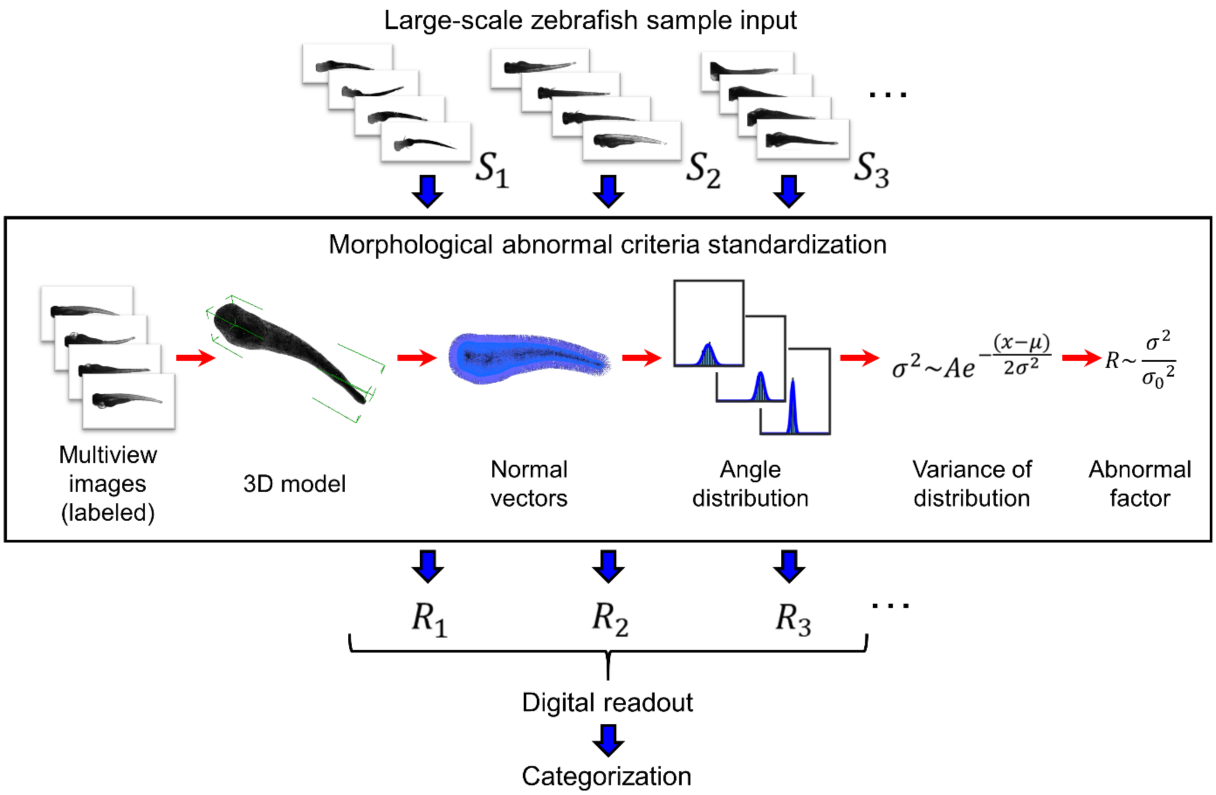

Supplementary Figure 15 | Flow chart of the abnormal level characterization and pipeline for morphological abnormal criteria standardization. The standardization process is indicated in the black frame that connects the empirical criteria with the numbers. As the process builds, large-scale zebrafish samples can be fed into the system to generate digitalized readouts for abnormal level quantification and used for categorization. The three categories for edema and tail curling are identified based on the statistical angle distribution for each category as: 1. Unaffected: $R \leq 1.5$, 2. Moderate: $1.5 < R \leq 4$, 3. Severe: $R > 4$.

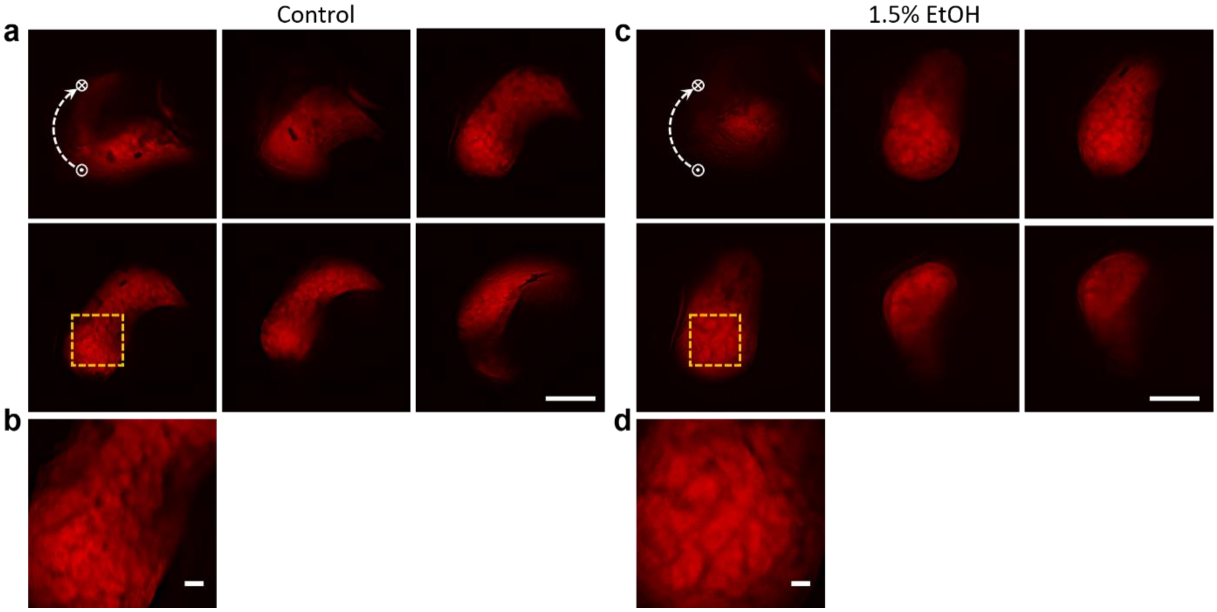

Supplementary Figure 16 | Imaging of the zebrafish larva liver via ART. a, Fluorescent image of the liver from the control group at different viewing angles. **b,** Magnified image of the liver. **c,** Fluorescent image of the liver from the ethanol-treated zebrafish larva. **d,** Magnified liver image. Three independent liver rotation experiments (n=3) were conducted in each group for visualization. Representative liver images from different angles were chosen to indicate the rotation motion. Scale bar: 100 μm for images at different angles, 20 μm for magnified images.

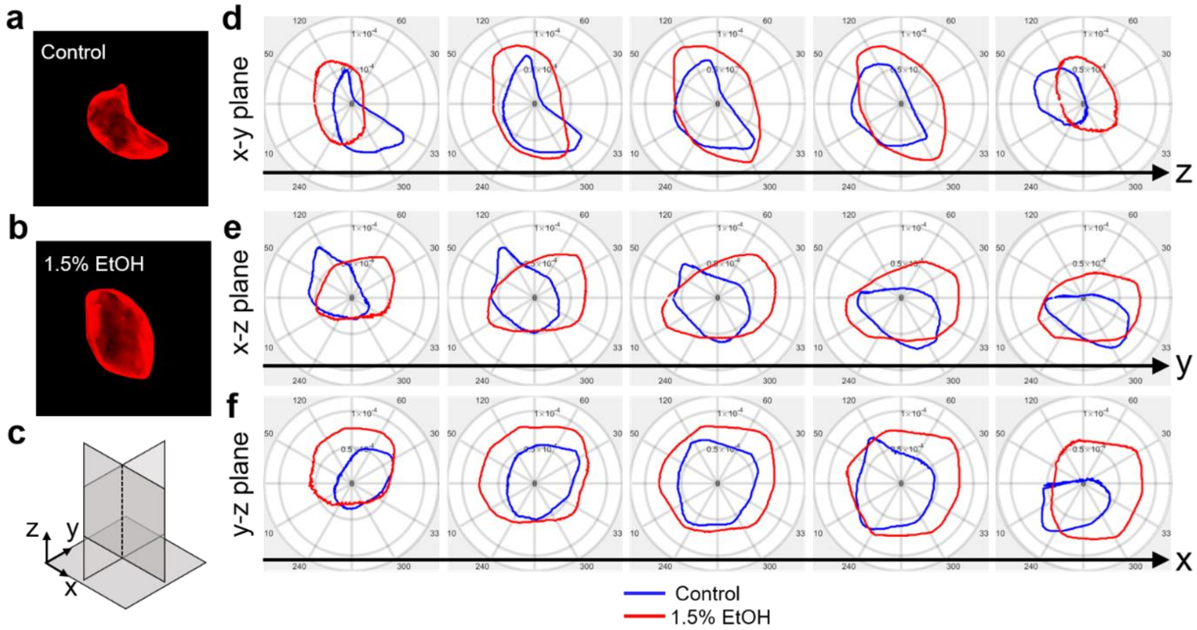

Supplementary Figure 17 | Outline contours from the projection of the typical reconstructed 3D model of a zebrafish liver in the control (a) and 1.5%-EtOH (b) groups. a, 3D model of a liver in the control group. **b**, 3D model of a liver in the 1.5%-EtOH group. **c**, Schematic shows the coordinate axis and the x-y, x-z, and y-z projection planes. **d-f**, Contours of the projection of the 3D model of the liver in (**a**) and (**b**).

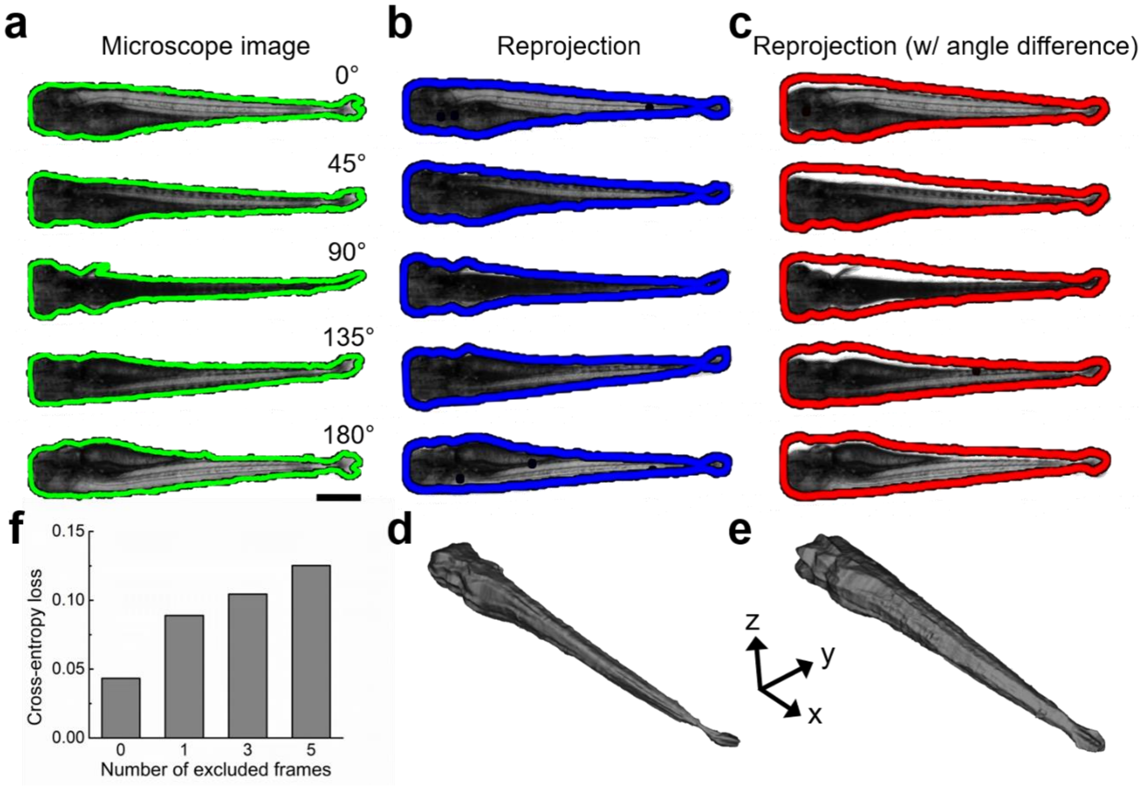

Supplementary Figure 18 | Comparison of the silhouette outlines between the microscope images, digital re-projection images, and the re-projection images with a known uneven angle distribution. a-c, The outlines of different re-projection cases as compared with the actual microscope images. In order to demonstrate the effect of incorrect angular alignment, we recorded a video of one rotation cycle and deleted several continuous frames in order to mimic an uneven, non-uniform rotation. **d**, The 3D model of the zebrafish reconstructed from the original image stack. e, The 3D model of the zebrafish reconstructed from video with frame removed in order to demonstrate angle unevenness. **f**, The relationship between the cross-entropy loss with respect to the number of the missing frames, as a measure of rotational uniformity.

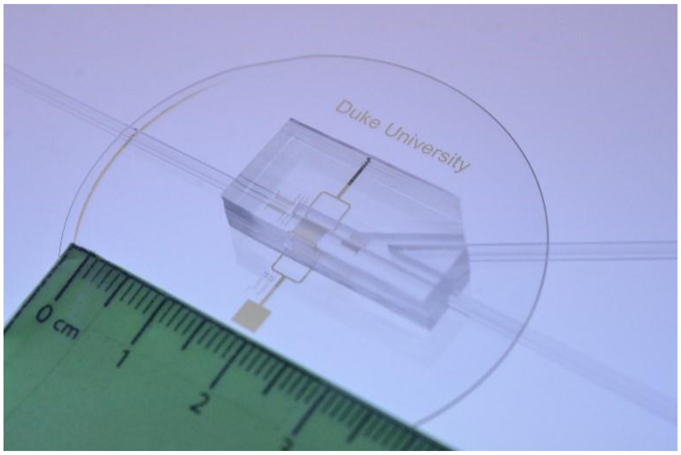

Supplementary Figure 19 | A photo showing the assembled acoustofluidic chip for rotational manipulation.

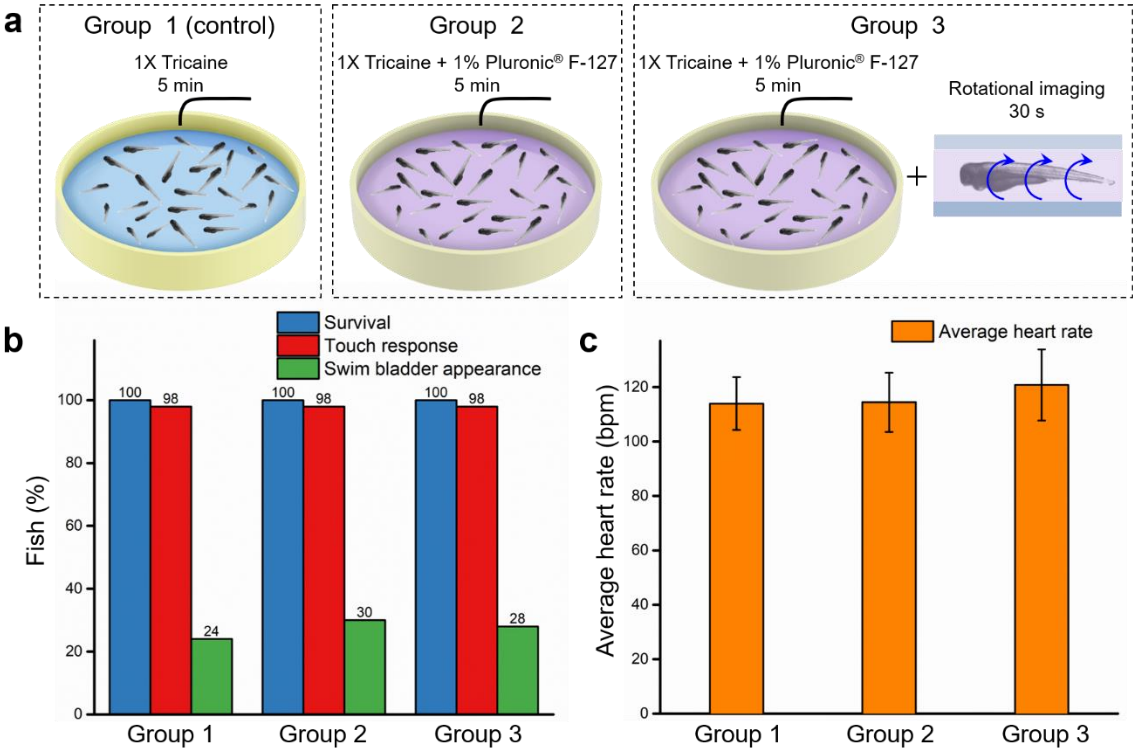

Supplementary Figure 20 | Assessment of zebrafish health. A health assessment was performed on three groups of 50 2CLIP zebrafish larvae to evaluate the viability of the ART platform. **a**, Schematics showing the test conditions of the three groups of 50 2CLIP zebrafish larvae studied in the health assessment. The three groups were treated as follows at 2 dpf: Group 1 (control group): 1X tricaine for 5 minutes; Group 2: 1X tricaine + 1% Pluronic® F-127 for 5 minutes; and Group 3: 1X tricaine + 1% Pluronic® F-127 for 5 minutes, and rotational imaged using the ART system for 30 seconds during this immersion time. After the treatment, the larvae were collected in cell strainers, gently rinsed with fresh DI water, and cultured for 48 hours. The health of the zebrafish larvae was assessed at their 4 dpf. **b**, The rate of survival, touch response, and swim bladder appearance at 4 dpf of each group (n=50 for each rate). **c**, Average heart rate of each group of 50 fish. The rate of survival, touch response, the appearance of swim bladder at 4 dpf, and heart rate in the Group 2 and Group 3 matched those of controls. The result of the health assessment shows that neither 5-min immersion in 1% Pluronic® F-127 nor 5-min immersion in 1% Pluronic® F-127 and 30-s rotational imaging using the ART platform causes obvious harmful effects to 2 dpf zebrafish larvae. Considering that the total Pluronic immersion time and the rotational imaging time in these tests is longer than our routine process, the health assessment demonstrates that routine processing via ART will barely affect the zebrafish larvae. n=50 for each group. Data are graphed as the mean ± SD.

Supplementary Tables

Supplementary Table 1 | Status of Acoustics, syringe pump, and valve in each mode of the flow system.

Step	Acoustics	Pump	Valve
Loading	On	On, withdraw	A-open/B-closed
Imaging	On	Off	A-open/B-closed
Unloading	On	On, push	A-closed/B-open

Supplementary Table 2 | Average time of each step.

Step	Time ± s.d.* (s)
Loading	5.5 ± 0.6
Rotation and imaging (1 cycle)	1.3 ± 0.3
3D reconstruction (body and organs)	39.6 ± 5.1
Unloading	2.9 ± 0.6

* Each time and standard deviation are averaged from $n = 20$.

Supplementary Note

In order to overcome the difficulties of generating on-demand vortex streaming with previous acoustofluidic methods, we proposed a unique design scheme which can generate single vortex streaming that is strong enough to manipulate zebrafish. With its unique design, our platform avoids the limitations imposed by the typical balance between vortex size, acoustic frequency and vortex strength. In our design, a fluidic channel with a square cross section is aligned parallel to the lateral side of the IDT (y axis), such that half of the channel overlaps with the fingers of the IDT (**Fig. 1a** and **Supplementary Fig. 1**). Since our device generates a streaming vortex through a combination of leaky-SAW induced body forces and the fluid interaction with the confining walls of the channel, the size of the vortex pattern is determined by width of the channel cross section (a) and the length of the IDTs (L). Thus, we are able to generate a strong and large (millimeter-scale) single vortex tube with a low input voltage (~10 Vpp) by increasing the frequency of SAWs, which significantly decreases the power consumption of the device. Moreover, the dimensions of the streaming vortex can be easily scaled for different applications.

Effect of width of the effective IDT area (e) to the streaming pattern. In this study, we proposed that the single vortex pattern can be generated by aligning the channel parallel to the lateral side of the IDT (y axis) with half of its width overlapping with the IDT area. Fixing the value of the channel width (a=1.34 mm), IDT length (L=3.15 mm), and frequency (f=23.9 MHz), we numerically explored the effect of the width of the effective IDT area (e) to the streaming pattern. As shown in **Supplementary Fig. 2**, a single vortex pattern, which extends along the length of the channel for the distance of the IDT length (L), can be generated when only a fraction of the channel overlaps with the IDT. And, the width of effective IDT area (e) affects the center of the vortex pattern (indicated by the red asterisks), as well as the stable rotation position of a zebrafish larva. When much less or much more than a half of the channel is placed on the IDT, the center of the single vortex is far away from the center of the channel in the y axis. In these cases, the rotation of the zebrafish larvae was predicted to be unstable because of the asymmetric streaming pattern and the possible resistance from the channel wall. By placing about half of the channel upon the IDT ($0.45a \leq e \leq 0.55a$), the center of the vortex streaming closely overlaps with the center of the channel in the y axis, where we predict the stable rotation axis of the zebrafish larvae will be. In this range, the distance of the 600-µm-wide larva to the channel wall will be about 350 µm, which allows the zebrafish larvae within the size range (<~850 µm for 5 dpf larvae) to be rotated with a low chance of contacting the channel wall. Thus, setting the ratio of the overlapping widths

of the IDT area (e) to the channel cross sectional width (a) to e=0.5a, which means that if half of the channel width was located upon the IDT the system can provide optimal performance for zebrafish larvae rotational manipulation.

Notably, through our numerical investigation, we found that this single vortex pattern can be generated for any size channel, as long as the "e=0.5a" condition is satisfied. Hence, the size of the vortex can be adjusted by scaling "e" and "a" proportionally. Moreover, the length of the vortex tube structure along the channel length is determined by the lateral side length of the IDT (L). These results imply that larger or smaller channels could be designed to handle organisms of various sizes, and the single vortex streaming pattern can be scaled to enable the acoustofluidic rotational manipulation of different vertebrates.

Effect of channel width (a). As proof that vortex tubes with adjustable sizes can be generated based on our chip design, we numerically and experimentally validated the formation of single vortex streaming within two channels with different sizes (a=0.8 mm and a=1.34 mm). And, we also tested their performance on rotational manipulation of zebrafish larvae. The results for the 1.34-mm and 0.8-mm channels are shown in **Fig. 2** and **Supplementary Fig. 3**, respectively. Both channels were placed over identical IDTs and aligned following the "e=0.5a" rule. The IDTs had an aperture width of W=2.24 mm, a lateral side length of L=3.15 mm, and were composed of 21 pairs of 37.5-μm fingers (**Supplementary Fig.1b**). **Fig. 2a-2c** shows the numerically simulated and experimental streaming pattern for the 1.34-mm channel with an input frequency of 23.9 MHz. 1-μm fluorescent particles were used to visualize the flow field in the top and bottom planes. From this experiment, we observed that the fluid was pulled toward the IDT along the bottom of the channel and pushed in the opposite direction along the top of the channel. This experimentally-observed fluid motion closely matched the numerical streaming patterns on the corresponding planes of the simulated channel. A similar phenomenon was found in the 0.8×0.8-mm^2 channel (**Supplementary Fig. 3a-c**). Thus, using this "e=0.5a" design rule, millimeter-scale single vortex patterns with centers aligned along the middle of the channel can be generated without changing the IDT configuration or applied actuation signals. And, the size of the vortex tube can be adjusted by scaling "e" and "a" proportionally for its width, and by scaling the IDT length "L" for its length.

We also observed that the rotational manipulation of the 5 dpf zebrafish larvae can be achieved when using the 0.8-mm channel. However, the narrower channel results in less liquid between the zebrafish larvae's body and the channel wall (**Supplementary Fig. 3d**), and hence less acoustic energy can be loaded into the smaller liquid volume for generating sufficient acoustic streaming. As a result, the rotation speed of the zebrafish larvae in a 0.8×0.8 mm^2 cross section

channel was approximately 65% slower than that in the 1.34x1.34 mm^2 channel (**Fig. 2e** and **Supplementary Fig. 3f**). Additionally, the reduced distance between the larvae and the channel wall increases the chance that the zebrafish will contact the wall and reduces the stability of the rotation. The smaller device also reduces the range of larvae sizes that can be manipulated within the channel (**Supplementary Fig. 3d,e**). Considering the efficiency of energy transfer and the shape/size variation across different zebrafish larvae, the channel with a 1.34×1.34 mm^2 cross section was chosen for the rotational imaging of zebrafish larvae in this work.

Supplementary Videos

Supplementary Video 1. Acoustofluidic rotational manipulation and imaging of a 5 dpf zebrafish.

Supplementary Video 2. The rotation speed of a 5 dpf zebrafish under 5 different input voltages.

Supplementary Video 3. The microscopic images and 3D reconstruction model projections of a *Tg(fabp10:DsRed; els3l: GFP)*^{gz12} 5 dpf zebrafish larva over one rotation cycle, including images of merged fields and exploded fields (bright field, DsRed, GFP).

Supplementary Video 4. Acoustofluidic rotational manipulation and imaging of three typical acute ethanol (EtOH) exposed zebrafish with morphological deformation.

Supplementary Video 5. Continuous imaging of acute ethanol (EtOH) exposed zebrafish liver.

Supplementary Video 6. Projections of zebrafish liver models in **Fig. 6c** from multiple angles.

Supplementary Video 7. Rotational motion of a zebrafish yolk within an intact chorion (0 dpf, prior to tail extension).
