# Supplementary material for: Acoustofluidic rotational tweezing enables high-speed contactless morphological phenotyping of zebrafish larvae
Source: Nat Commun. 2021 Feb 18;12:1118. doi: 10.1038/s41467-021-21373-3 (PMC7892888; doi:10.1038/s41467-021-21373-3)
Supplement: Supplementary file 2 — Description of Additional Supplementary Files [file 41467_2021_21373_MOESM2_ESM.pdf]

Title: Supplementary Video 1.
Description: Acoustofluidic rotational manipulation and imaging of a 5 dpf zebrafish.

Title: Supplementary Video 2.
Description: The rotation speed of a 5 dpf zebrafish under 5 different input voltages.

Title: Supplementary Video 3.
Description: The microscopic images and 3D reconstruction model projections of a *Tg(fabp10:DsRed; els3l: GFP)*^{gz12} 5 dpf zebrafish larva over one rotation cycle, including images of merged fields and exploded fields (bright field, DsRed, GFP).

Title: Supplementary Video 4.
Description: Acoustofluidic rotational manipulation and imaging of three typical acute ethanol (EtOH) exposed zebrafish with morphological deformation.

Title: Supplementary Video 5.
Description: Continuous imaging of acute ethanol (EtOH) exposed zebrafish liver.

Title: Supplementary Video 6.
Description: Projections of zebrafish liver models in **Fig. 6c** from multiple angles.

Title: Supplementary Video 7.
Description: Rotational motion of a zebrafish yolk within an intact chorion (0 dpf, prior to tail extension).
